# Microplastic contaminants potentially distort our understanding of the ocean’s carbon cycle

**DOI:** 10.1371/journal.pone.0334546

**Published:** 2025-10-13

**Authors:** Luis E. Medina Faull, Gordon T. Taylor, Steven R. Beaupré

**Affiliations:** School of Marine and Atmospheric Sciences, Stony Brook University, New York, New York, United States of America; University of Maryland Center for Environmental Science, UNITED STATES OF AMERICA

## Abstract

Direct observations confirm that admixtures of sedimentary organic matter (SOM) and microplastics (MPs) are fully oxidized during Elemental analysis (EA), with measured carbon yields, % carbon [%C], C:N ratios, stable- (δ^13^C) and radiocarbon (Δ^14^C) abundances consistent with predictions for SOM samples intentionally contaminated with plastic. As an example, MPs would comprise ~40% of all carbon atoms measured via EA in a 100 μg SOM sample (1% OC by mass) that has been contaminated with only 1 μg of polyethylene (PE = 77% C by mass). This MP contamination, amounting to just 1% of the total sample mass, would lower the sample’s Δ^14^C by 258‰ to −622 ‰, lower the sample’s δ^13^C by −3.65‰ to −25.22‰, and overestimate its conventional ^14^C age by ~4000 years. Moreover, this 1% MP contamination would imply a terrestrial source contribution of ~ 60% instead of the 20% for an uncontaminated SOM sample. Our results illustrate how these errors scale predictably with MP contamination level and dominant polymer types. While large errors might be recognized as outliers and scrutinized, even small levels of contamination (e.g., 0.1% by mass) can introduce significant but subtle errors that could go unnoticed (e.g., Δ¹⁴C error of −30‰). Most carbon biogeochemistry studies do not routinely recognize the presence of MPs in environmental samples, despite the ubiquity of MP in the ocean and their potential impact on measurements. Consequently, MP contamination either naturally-occurring in field samples or introduced while sampling and processing will necessarily lead to errors in organic matter characterization, source apportionment, and estimates of conventional ^14^C ages.

## Introduction

Microplastics (MPs) are defined as any plastic particles ≤ 5 mm in diameter and they have been found in all marine environments [1,2]. These carbon-rich materials (≥ 70% of mass) can enter marine food webs via deposit or suspension-feeding organisms [3]. They also can play an important role as substrate for microbial biofilms [4,5]. In addition, marine aggregates may be important sites for interactions between MPs and particulate and dissolved OM [6]. Marine aggregates can incorporate not only plankton debris into their matrix but also clay and other non-organic particles [7]. Therefore, MPs are also likely to be incorporated into marine aggregates and potentially measured as some proportion of the biogenic carbon pool produced in the ocean and exported to depth [8–11].

In the laboratory, natural OM environmental samples are commonly analyzed by thermal decomposition, such as pyrolysis or gasification in various instruments [12–14]. Elemental analyzers (EA) and other common methods of dry combustion oxidize all reduced organic carbon atoms to CO_2_ [15]. Due to overlapping thermal combustion ranges of natural organic matter and plastics, MP contaminants coexisting with marine biogenic OM are expected to combust together during routine analyses. Thermal decomposition of plastics and their pyrolysis products are well-characterized [16]. For example, synthetic polymers like polystyrene (PS), polypropylene (PP), and polyvinylchloride (PVC) will pyrolyze at temperatures as low as 500–800° C [17]. However, absent are empirical determinations of co-combustion of plastic particles and biogenic OM in an EA (>960°C), which is commonly used to determine abundance, elemental stoichiometry, and isotopic composition of water column particulate organic matter (POM), sedimentary organic matter (SOM), and various concentrated forms of dissolved organic matter (DOM).

A few research groups have postulated that unrecognized MP contaminants could lead to misinterpretations of combustion-based organic matter measurements [8–11,18,19]. Inadvertent MP inclusion could directly lead to poorly-constrained errors in assessments of biogenic OM inventories, elemental stoichiometries, and isotopic compositions in environmental samples. Consequently, computation of reaction rates, biogeochemical fluxes, radiocarbon ages, residence times, provenance and source apportionments, and biogeochemical budgets are all potentially aliased by MP contaminants. To date, empirical testing of this proposition has not appeared in the literature.

In light of this knowledge gap, we first tested the hypothesis that EA data are quantitatively aliased by MP contamination. To do so, we prepared a range of MP and natural SOM admixtures to quantify the influence of MP contamination on routine OM analyses via EA. We demonstrate that plastic-derived and natural organic carbon are co-oxidized to completion. Then, we assessed the consequences of MP-derived artifacts by investigating to what extent other important carbon cycle measurements are affected by MP inclusion in routine measurements. For example, isotopic analyses are fundamental tools, widely used to characterize sources, diagenetic processes, and ages of marine biogenic OM. In particular, radiocarbon (^14^C) dating has been used to calculate ages of bulk DOM, POM and SOM pools throughout the world’s oceans in order to identify OM sources and sinks, and constrain ocean basin-wide residence times of OM inventories [20,21]. Similar to EA, techniques used to determine isotopic abundances of biogenic OM based on the analysis of bulk samples are likely blind to MP contamination. ^14^C dating will be biased to varying degrees by the presence of MPs since most plastics are manufactured from ancient petroleum, which is entirely devoid of ^14^C.

Our results confirm that MPs collected intentionally or unintentionally with a SOM sample contribute commensurately to the natural carbon signal and hence alias EA measurements. In addition, we demonstrated that even a small proportion of ^14^C-depleted MPs added to ^14^C-modern carbon in ocean inventories will artifactually age natural carbon pools and erroneously lead to the conclusion that OM pools are more chemically refractory than they actually are.

## Materials and methods

Experiments were conducted at the National Ocean Sciences Accelerator Mass Spectrometry (NOSAMS) facility Woods Hole Oceanographic Institution, under their Research Initiative program. The first experiment tested whether conventional methods used to quantify OM and its isotopic composition from marine samples are affected by the inclusion of plastic-derived carbon. Estuarine sediment (see below) was used as the natural, background SOM pool due to its higher volumetric carbon content compared to suspended POM and DOM in seawater. Polyethylene (PE) fragments were mixed with sediment samples to produce a range of weight percentages of MPs to be analyzed by EA and accelerator mass spectrometry (AMS). In addition, a second experiment was conducted at the Geosciences Department, Stony Brook University using polystyrene (PS) fragments to demonstrate that other polymers are similarly fully oxidized via EA. The methodology for the second experiment can be found in supplementary information.

We then compared measured and predicted masses (m), weight-weight proportions of carbon per mass of plastic or organic matter (p, where 0 < p < 1, which is expressed as percent after multiplying by 100), and fractional isotopic abundances (*f*) of plastic (*f*_p_), natural OM (*f*_om_), and their admixtures (*f*_*Σ*_). Key equations illustrating important concepts or used in computations and their derivations are presented as supplementary information.

### Experimental design

The first experiment was designed to test whether a realistic range of MP particle sizes (S1 Table, supplemental information) affects oxidation efficiency in an EA and whether MP particle size affects the degree to which plastic loadings induce measurement errors. EAs are calibrated to accommodate different masses of carbon based on several factors: 1) their intrinsic design, 2) the selection of standard materials, including their chemical composition and physical properties, 3) predicted carbon yields that determine chosen ranges of the standard’s masses, and 4) volumes of the standards along with associated sample cup sizes, which are influenced by density and carbon content of the standards. Therefore, accuracy and precision of analyte quantification can suffer if the analytes’ properties depart significantly from those of the standard or are insufficiently compatible with the instrument’s calibrated performance, e.g., yielding peaks that are too large (saturation and clipping). For these reasons, combustion efficiency of MPs may also depend on particle size, surface area to volume ratio, composition (i.e., %C), and interference with the sample matrix. For this experiment, commercial black polystyrene (PS) fragments in 3 different size fractions (60–125, 125–250, and 250–600 μm) were analyzed via EA at the Geosciences Department, Stony Brook University. In addition to pure end members containing only sediment or only PS particles, PS and marine sediment admixtures were combusted via EA. For the end members, triplicate samples were analyzed to establish the %C of both components (S2 Table, supplemental information).

Finally, we tested whether conventional methods used at the National Ocean Sciences Accelerator Mass Spectrometry (NOSAMS) facility to quantify OM and its isotopic composition from marine samples are affected by the inclusion of plastic-derived carbon. PE and marine sediment admixtures in addition to pure end members containing only sediment or only PE particles were analyzed for ^14^C by accelerator mass spectrometry (AMS) and for ^13^C by Isotope Ratio Mass Spectrometer (IRMS).

### Plastic samples: Source and preparation

Commercial PS black pellets and PE yellow fragments, approximately 5 mm in diameter, were obtained from Domino Plastics Company Inc, NY, US. Once cleaned with 70% ethanol, pellets were frozen at −80° C to facilitate their breakup while grinding to smaller particles using a stainless steel blender (Waring Inc.). The mixture then was size-fractionated sequentially with analytical stainless-steel sieves (DIN 4188, Retsch GmbH, Germany) into particles with nominal diameter ranges of 60–125, 125–250, and 250–600 μm. PS and PE were selected because of their high carbon contents (~90 and ~70% C) and environmental prevalence. Notably, PE and PS are among the seven most commonly produced plastic polymers, are commonly used in consumer goods, and are ubiquitous in the environment, commonly present in seawater and marine sediments [22–24].

### Sediment samples: Source and preparation

Sediment samples were collected in 1993 at station P, one of 19 Long Island Sound Study sites [25], located in central Long Island Sound (41° 10.03´ N, 72° 57,43´W) at 16 m water depth. Sediments were retrieved using metal sediment box cores (depth in core 230–240 cm) and frozen (−20°C) until the present analysis. Once thawed, 250 g sediment subsamples were homogenized in a porcelain mortar for several minutes. Carbonates and dissolved inorganic carbon were removed by titration with a 10% HCl solution. After reaching a pH of 2–2.5, samples were dried in an oven (60°C) for 48 h and stored as a fine powder at −20°C until further use. This procedure ensured that CO_2_ produced by the samples was derived from biogenic organic carbon and plastic particle amendments [26].

### Carbon content and isotopic composition

Carbon content, stable isotope and radiocarbon of SOM, PE and their admixtures were measured at the NOSAMS facility. Detailed information of sample requirements, preparation, measurement, and the procedures for ^13^C and ^14^C measurements, δ^13^C, Δ^14^C, and age calculation are available at the NOSAMS website (https://www2.whoi.edu/site/nosams/resources/methods/). Briefly, all samples were combusted in Elemental Analyzer (Elementar, vario ISOTOPE Select), which directly quantified the %C, %N, and C:N values. CO_2_ generated from each sample by the EA was split into two aliquots, one was used to measure ^13^C offline in a Elementar, isoprime precisION Isotope Ratio Mass Spectrometer (IRMS), and the second was reduced to graphite on an iron catalyst [27,28]. The graphite was then pressed into targets, which were analyzed for ^14^C by accelerator mass spectrometry (AMS) along with primary and secondary standards, ^14^C standards and process blanks.

## Results and discussion

### Microplastics particle size effect

Triplicate PS samples from three different size fractions (60–125, 125–250, and 250–600 µm), were combusted to test the hypothesis that EA combustion efficiency is unaffected by plastic particle size. Total PS carbon content among all samples varied from 0.33 to 0.52 mg C per sample (S1 Table). The % C values (89.5–95.5%) were not significantly different between the various PS size fractions (S1 Table; p > 0.05; Kruskal-Wallis test of significance). Furthermore, % C values did not vary with individual sample masses. Therefore, PS combustion efficiency was statistically independent of particle size and sample mass over the range examined. Note that the average % C value of our PS samples (91.0 ± 1.1 mass % C, n = 12) observed by EA was only slightly lower than the theoretical value of pure PS (92%), but within analytical uncertainty. We postulate that observed variations resulted from the black dye additive in these PS particles. Regardless, PS carbon mass % was significantly higher than that of SOM (e.g., ~ 1 mass % C in Long Island Sound sediments).

### Microplastics, an uncharacterized fraction of the ocean’s organic carbon inventory

Total masses and MP contributions to the two sets of sediment samples amended with either PS or PE particles appear in S2 and S4 Tables, respectively. By incrementally adding small masses of carbon-rich PS (1.3–5.4 mass % additions) or PE (0.14–8.5 mass % additions) to sediment samples, apparent carbon content (C mass/total mass) of SOM- PS admixtures increased by 65–634% C and for SOM-PE mixtures between 24 and 717% C relative to unamended SOM (S3 and S5 Tables).

**Fig 1:**
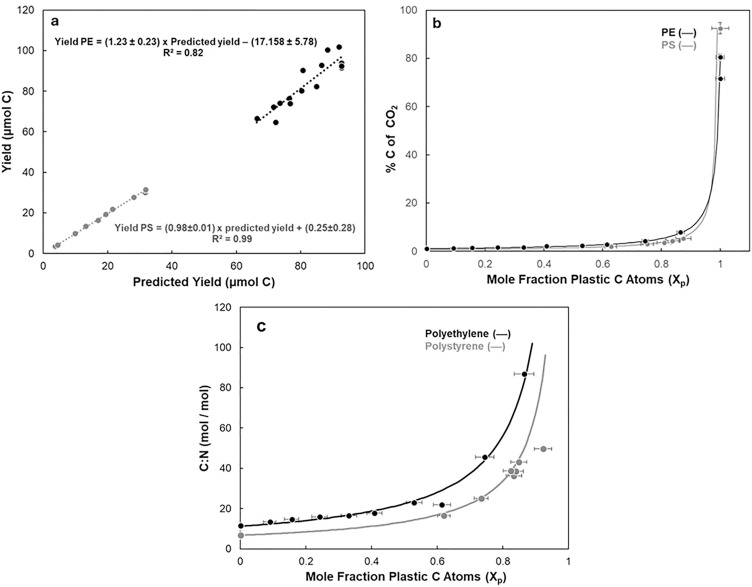
Elemental analysis (EA) measurements of sedimentary organic matter (SOM) mixed with varying amounts of PS (grey) or PE (black). **a)** Mean measured vs. predicted yields of carbon dioxide. **b)** Mean carbon content (% C) of admixtures as a function of C mole fraction of PS (grey) or PE (black) in admixtures. Errors bars plotted for all measurements but are smaller than most data points. **(c)** Measured (circles) and predicted (lines, Eq. S44) effects of MP contamination on SOM C:N stoichiometry as a function of C mole fraction of PS (grey) or PE (black) in admixtures. Sediment PS (grey) and PE (black) amendments are equivalent to 1.3–5.4 and 0.14–8.5 mass % additions, respectively. C:N error bars (± 1 standard error (SE)) are smaller than data points.

To confirm the strength of the relationship between actual and predicted yields, a least-squares linear regression analysis was applied to both admixtures (Fig 1a). Observed C yields did not significantly differ from those predicted (p > 0.05; Kruskal-Wallis). The two estimates were highly correlated (PS r^2 ^= 0.99 and PE r^2 ^= 0.82) with a slope of 0.98 ± 0.01 for PS and 1.23 ± 0.23 for PE. These relationships are consistent with non-selective and nearly complete co-combustion of both plastic and SOM. Furthermore, this confirms that even minor MP contamination levels lead to artificially high C yields and thus higher inferred %C in natural organic materials analyzed by EA. The non-linear relationship between % C in sediment admixtures (*p*_Σ_) and mole fractions of MP contaminants (X_*p*_) illustrates that increasing the mole fraction of PS or PE with small MP mass additions (0.14–8.5%) leads to an outsized inflation of apparent %C in SOM samples (Fig 1b).

Unintentional inclusion of MP particles and their leachate in fundamental studies of organic matter inventories, movement, transformation, elemental stoichiometry, and storage in important ocean reservoirs may lead to misconceptions. For example, carbon and nitrogen content in marine OM samples measured since at least 1972 by EA [29], are routinely used to calculate elemental compositions of marine plankton in surface waters [30]. When compared to the Redfield ratio (106C:16N:1P), particulate C:N stoichiometry provides insights into a variety of biogeochemical processes, such as phytoplankton productivity, detrital decomposition dynamics, and loadings, and rates of nitrogen fixation and loss.

The sensitivity of measured C:N ratios on the mole fraction of the MPs in SOM is presented in Fig. 1c. C:N values increase as predicted when assuming that MP particles (PS and PE) are quantitatively co-combusted with the SOM. Solid lines are predicted values, which were calculated independently of the data and yield the same results within analytical uncertainty. This exercise illustrates that in the absence of MPs, the C:N ratio was 6.66 for SOM-PS and 11.23 for SOM-PE or essentially very close to the Redfield ratio. However, with modest MP contamination of that same SOM (0.4 mol C of PS or PE per total mol of C in each mixture), the apparent C:N now becomes ~11.4 and ~18.71, respectively.

Unprecedented C:N signatures have been reported for Arctic Ocean surface waters [31]. These unusual values at some stations may have resulted from methodological artifacts, such as low signal to noise ratio (POC signals less than twice that of the filter blank) or C:N ratios being potentially influenced by adsorption of dissolved organic carbon (DOC) onto filters [31]. Likewise, carbon-rich transparent exopolymeric particles (TEP) could contribute to unusually high C:N ratios in some areas of the ocean [32,33]. An alternative explanation for observed anomalously high C:N ratios in some instances may actually be unrecognized MP contamination. A modest mass of MP contaminants could have contributed outsized proportion of carbon in suspended and sinking POM in the ocean.

Additionally, we note that MPs and plastic microfibers are pervasive in shipboard and lab settings (including synthetic fabrics) and may inadvertently be introduced while processing samples for analysis and escape the analyst’s notice. The present study confirms that carbon from unrecognized plastic contaminants is quantitatively measured by the Elemental Analysis method. Our experiments illustrate that C:N ratios in MP-SOM admixtures vary non-linearly but predictably with the mole fraction of carbon atoms derived from MP contamination. Consequently, POM samples contaminated with even small amounts of MPs could result in significantly biased C:N measurements and hence yield misleading elemental stoichiometries. Thus, MP contamination may have far-reaching implications for current assessments of carbon cycling, transport and elemental stoichiometry in the ocean and those from the recent past.

### Microplastic contaminants induce significant biases in carbon isotope signals

**Fig 2:**
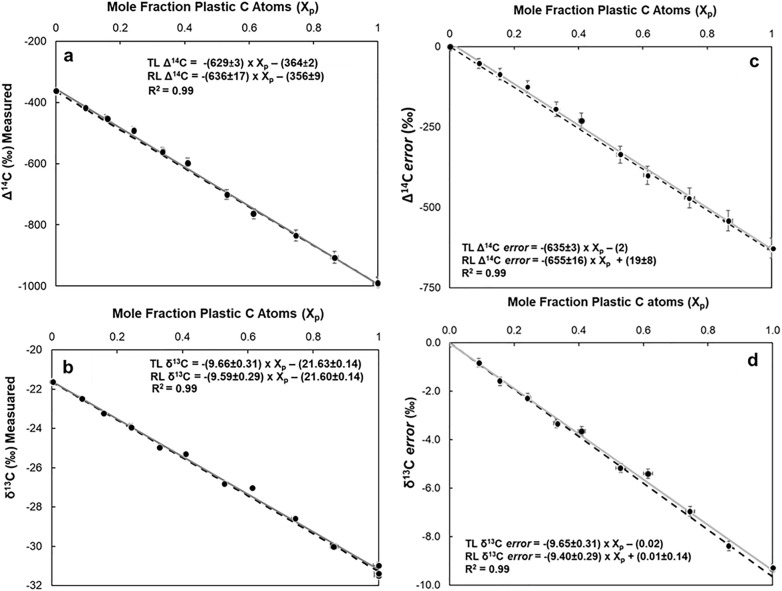
Effects of MP contamination on carbon isotope observations in sediments enriched with 0.14–8.5 mass % of PE. (a) Mean observed Δ^14^C values (circles ± 1 S.E.) compared to the fraction of carbon atoms from MP (X_p_) in admixtures. (b) same as (a) for δ^13^C. Error bars (± 1 S.E.; S6 Table) were smaller than most data points. (c) Mean computed Δ^14^C errors (circles + 1 S.E.) compared to MP carbon molar contribution to SOM (X_p_) in admixtures. Errors = measured value – expected value of uncontaminated sediment. (d) same as (c) for δ^13^C. In all panels, solid grey lines are least-squares linear regression results for all measured values (“RL” in equations). Dashed lines are the theoretical relationships between measurements and mole fraction of C atoms contributed by PE (“TL” in equations).

Incremental additions of the carbon-rich PE to sediment samples decreased measured Δ^14^C, and δ^13^C values in admixtures from −416 to −905‰ (Fig 2a) and −22.46 to −30.02 ‰ respectively (Fig 2b, see also S6 Table). Consequently, MPs inadvertently collected and analyzed with routine particulate organic matter (POM) samples for isotopic analysis led to biased Δ^14^C and δ^13^C values because petroleum-based plastics have isotopic signatures distinctly different from those of modern autochthonous OM [34].

Furthermore, the Δ^14^C and δ^13^C errors (i.e., measured value – expected value for uncontaminated sample; Fig 2c and 2d) in these observations follow trends expected from conservation of mass (dashed black lines in Fig 2c and 2d), decreasing with the incremental additions of ^14^C and ^13^C-depleted PE to sediment samples. These errors are directly proportional to the mole fraction of plastic carbon contamination, and to differences between Δ^14^C and δ^13^C signatures of the autochthonous OM and plastic carbon.

**Fig 3:**
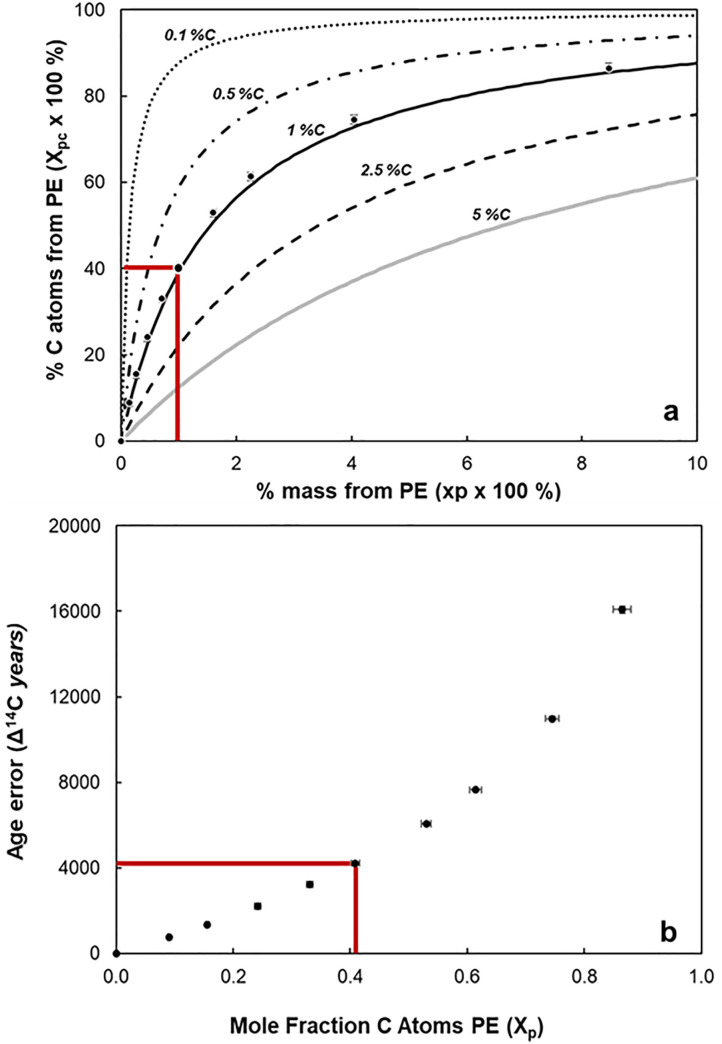
Relationship between mole, mass fraction and apparent 14C age error. (a) Mole and mass fraction of PE in admixtures. Lines represent the theoretical relationship (Eq. S55) for hypothetical sediments samples with a wide range of biogenic C content (0.1, 0.5, 1, 2.5 and 5%C) found in a variety of marine environments. The red box illustrates that a sediment with 1% biogenic OC contaminated with only 1% MP by mass would have ~40% of its C atoms originating from PE. Thus, a 100 μg sediment sample (1% C) with only 1 μg of plastic (PE 70.5% C) would have 40% of C atoms from plastic. Circles represent mean EA measurements (± 1 S.E.) of LIS sediment mixed with PE. (b) Apparent ^14^C age error measured as a function of MP mole fraction of SOM carbon. The red box indicates sedimentary organic carbon contaminated with only 1% by mass of MP, or equivalently 40% of plastic carbon atoms, could have an age error of ~ 4000 ^14^C years.

Marine sediments have been recognized as an important sink for MP [35–37], with recent estimates suggesting that 22 Tg of MP (10 µm to 2.5 mm in diameter) are deposited annually on and/or buried in these environments [35]. Moreover, estimates indicate that carbon from MP could contributes up to 1.2% to the sediment carbon pool in coastal wetland sediments [38]. In addition, samples from sediments traps in deep waters (>2000 m) showed that carbon contribution to POC from MP could vary from between 1.5% and 5% [10,11,39,40]. Small apparent masses of plastic contamination can constitute a large proportion of the total number of carbon atoms in a natural particulate sample (Fig 3a).

If, for example, only 1% C by mass (S5 Table) of PE (δ^13^C_p_ = −32.6 ± 0.14 ‰, Δ^14^C_p _= −993 ± 2.6 ‰ and %C = 77 ± 1) contaminated our SOM sample (δ^13^C_om_ = −21.65 ± 0.14 ‰, Δ^14^C_om _= −364 ± 2 ‰ and % C = 1.12 ± 0.03%), then MP contamination would account for ~40% of the total C atoms measured via EA ($X_{p}=\text{0}.\text{4}\text{0}\text{9}\;(\text{4}\text{0}\%),$ Fig 3a). This would have significant implications for interpretation of isotopic composition, fractionation, and age. As an example, our central basin of LIS had a typical surface sediment ^14^C age of ∼2900 years, with a range from ∼2000–4000 years [41]. Therefore, 1% by mass of PE contamination of this sediment would lead to a Δ^14^C error of ~ −258 ‰ which lowers the SOM Δ^14^C value from −364 ‰ (no contamination) to −622‰. This would erroneously overestimate conventional ^14^C age by ~4000 years (Fig 3b), artifactually increasing the ^14^C age to ~ 6900 years. This level of contamination (e.g., 1 μg MP in 100 μg of sediment) would be difficult to detect without careful screening. Thus, even 1% of MP contamination by mass in the sediments will artificially age the natural SOM pool, which is a direct indicator of its lability, and therefore would also artifactually make the natural carbon pools appear more refractory.

**Fig 4:**
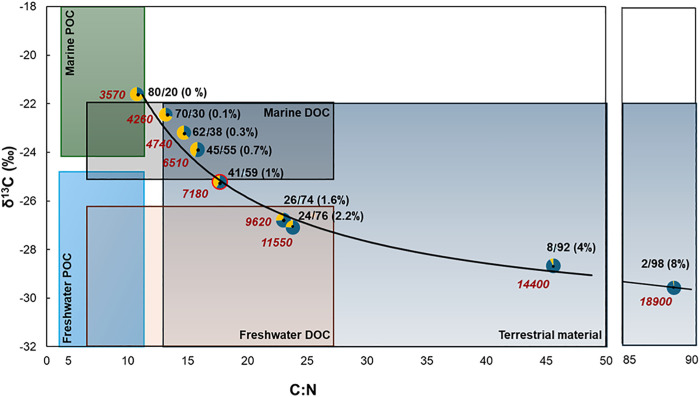
Relationship between δ^13^C, C:N, and ^14^C age for our PE-sediment admixtures. Colored boxes indicate typical compositions (δ^13^C) of major C reservoirs, including particulate organic carbon (POC), dissolved organic carbon (DOC), and terrestrial material in the environment (after [44]). Pie charts appear at the measured δ^13^C and C:N values of the SOM/PE admixtures used in this study, with the percent mass of PE indicated in parentheses. Pie chart colors and numbers indicate the apparent contribution of marine (yellow) and terrestrial (blue) carbon that would be calculated for these admixtures based on a two-component mixing model [42]. The red-outlined pie chart corresponds to just 1% MP contamination by mass. Red numbers correspond to apparent radiocarbon age in conventional ^14^C years. The black curve represents the predicted relationship between δ^13^C and C:N for these admixtures (Eq. S77).

Moreover, a 1% MP contamination by mass in SOM samples (1 µg of C from MP in 100 µg of SOM) would artificially increase C:N values from ~ 8, essentially the Redfield ratio, to an apparent C:N ~ 18 (Fig 1c; S5 Table). This apparently trivial amount of plastic in the SOM samples would decrease the δ^13^C from −21.65 ‰ (uncontaminated SOM) to −25.22 ‰ (δ^13^C error = −3.65 ‰). This error exceeds the uncertainty of high precision IRMS measurements (~0.02 ‰) by nearly two orders of magnitude, leading to increased uncertainties in measurements, artifactual isotopic fractionation factors, and/or erroneous source apportionments. Applying a simple mixing model [42,43] we calculated the apparent δ^13^C of our SOM contaminated with an increasing amount of PE. Fig 4 illustrates how increasing MP contamination levels can utterly change the apparent composition and provenance of natural OM. For example, again contaminating our SOM sample with only 1% MP by mass would significantly increase the apparent contribution of terrestrial material from ~20% to 60% (Fig 4, pie chart outlined in red). These errors would provide misleading evidence for the OM provenance, potentially leading to non-trivial errors in estimates of transport and mixing rates, respiration and productivity rates, or the spatial and temporal dynamics of the SOM in coastal or deep sediments.

These findings lead to two key conclusions. First, even modest contamination by microplastics can significantly alter the apparent composition and provenance of sedimentary organic matter (SOM). For example, in our assayed SOM sample, just 3% MP contamination by mass would account for approximately 74% of all measured carbon atoms. This results in a Δ¹⁴C offset of ~ –470‰, decreasing the SOM Δ¹⁴C value from –364‰ (uncontaminated) to –834‰. This shift would artifactually increase the conventional radiocarbon age estimate by ~10,830 years, producing an erroneous age of ~14,400 years (Fig 3b). At this contamination level, the δ¹³C value would be skewed to –28.59‰ (δ¹³C error = –6.923‰), with a C:N ratio of 45.6, yielding an apparent terrestrial contribution of ~92% (Fig. 4). Ideally, such extreme deviations may be recognizable as outliers in a broader geochemical context and flagged for further scrutiny.

However, even lower levels of MP contamination (e.g., 0.2% by mass, representing ~9% of total carbon in the sample) can introduce substantial errors. This degree of contamination results in a Δ¹⁴C shift of ~–52‰, reducing the SOM Δ¹⁴C value from –364‰ to –416‰. The corresponding radiocarbon age would be overestimated by ~690 years, yielding an apparent age of ~4,260 years (Fig. 3b). Additionally, δ¹³C would shift to –22.46‰ (δ¹³C error = –0.83‰), with a C:N ratio of 13.3 and an inferred terrestrial contribution of ~30% (Fig. 4). These errors, while smaller, may be subtle enough to go undetected. This analysis highlights the importance of rigorously evaluating plastic contamination in sediment geochemical analyses.

The quantity of plastic waste entering the ocean is predicted to increase by up to one order of magnitude by 2100 [10,45]. Scenarios for climate change are based on our abilities to model and predict the consequences of past and present human-generated stressors such as the plastics crisis. The accuracy of these models depends, among other factors, on reducing inherent data uncertainties used in their implementation. This makes determination of possible effects of micro carbon-rich particles on models’ uncertainties a critical task. Based on our results, we argue that many measurements and models of carbon cycling and elemental stoichiometry are very sensitive to, and unwittingly influenced by MP contamination. This necessarily leads to potential biases in carbon budget calculations and their implications for global climate. In addition, review and possibly revision of practices used to measure OM in oceanic reservoirs and elsewhere are critical to avoid MP contamination during sampling, storage, processing, and analysis of OM samples.

Finally, we note that MP contaminant effects may vary among different combinations of synthetic polymers with different %C, additives, plasticizers and pigments (e.g., polycarbonate. polyethylene terephthalate, polypropylene, etc.), natural OM samples (sediments, water, soil, snow, ice), and analytical methods (pyrolysis, optical or chemical). However, our results from EA-IRMS combustion experiments demonstrate that observed C yields and %C values agree with predicted values for intentionally contaminated samples. Observed yields are not influenced by MP particle size nor the mass contribution of plastic contamination. Importantly, carbon recoveries as CO_2_ were essentially 100% (within analytical uncertainty) among MP:SOM admixtures ranging from 0 to 100% MP. From this we conclude that carbon yields are commensurate with the level of plastic contamination across the range tested.

## Conclusions

Overall, we established that all bulk chemical characteristics of SOM quantified by EA-based analyses (C yield, %C, %N, C:N, ^13^C, and ^14^C) will be biased in direct proportion to the amount (and type) of MP contamination. Even low levels of MP contamination can lead to non-trivial errors in estimates of C inventories, elemental stoichiometry, source apportionments, inferred biogeochemical reaction pathways (e.g., based on isotopic fractionation and models), and chemical reactivity/ turnover times. In addition to being mindful of MP contamination inherent to most modern environmental OM pools, our results underscore the need to re-evaluate best practices for processing OM samples for carbon analysis. Microfibers from clothing and plastic particles from sampling, storage, and processing gear can all enter samples unnoticed and become part of measured carbon inventories. Additionally, it is important to consider that other applications relying on carbon isotopes that non-selectively combust organic matter with MP contamination are probably subject to the same biases, e.g., radiocarbon dating of artifacts.

## Supporting information

S1 Text Expected relationships between the amount of plastic contamination in organic matter samples and their measured bulk properties. (DOCX)

S2 Text Methodology for the EA experiment conducted at the Geosciences Department, Stony Brook University. (DOCX)

S1 Table Combustion performance of EA-IRMS for polystyrene (PS) microparticles within three different size fractions. Pa (1,2,3), Pb (1,2,3) and Pc (1,2,3) are triplicate polystyrene samples of different size fractions. Sample mass (MP mass) was measured by microbalance.
(DOCX)

S2 Table Masses of pure Polystyrene (PS) microplastics, pure sediment, and their admixtures, and the % plastic by mass in each sample. Uncertainties in % plastic mass were propagated from the plastic mass and total mass assuming their uncertainties were ± 1 μg.
(DOCX)

S3 Table Elemental composition of the pure Polystyrene (PS) microplastics, pure sediment, and admixture samples listed in Table S2, as measured by EA-IRMS. (DOCX)

S4 Table Masses of pure Polyethylene (PE) microplastics, pure sediment, and their admixtures, and the % plastic by mass in each sample. Uncertainties in % plastic mass were propagated from the plastic mass and total mass assuming their uncertainties were ± 1 μg.
(DOCX)

S5 Table Elemental composition of the pure Polyethylene (PE) microplastics, pure sediment, and admixture samples listed in Table S4, as measured by EA-IRMS. (DOCX)

S6 Table Carbon isotopic data measured at the National Ocean Sciences Accelerator Mass spectrometry (NOSAMS) for pure PE microparticles, admixtures, and sediment. (DOCX)
