# Supplementary material for: Microplastic contaminants potentially distort our understanding of the ocean’s carbon cycle
Source: PLoS One. 2025 Oct 13;20(10):e0334546. doi: 10.1371/journal.pone.0334546 (PMC12517520; doi:10.1371/journal.pone.0334546)
Supplement: S1 Text — (DOCX) [file pone.0334546.s001.docx]

Supporting Information for

**Microplastic contaminants potentially distort our understanding of the ocean's carbon cycle**

Luis E. Medina Faull* (ID 0000-0002-9425-986X), Gordon T. Taylor (ID 0000-0002-6925-7571), and Steven R. Beaupré (ID 0000-0001-6964-1058)

School of Marine and Atmospheric Sciences, Stony Brook University, New York, U.S.A

# Introduction

This study examines potential errors imposed by microplastic (MP) contamination on measurements of natural organic matter (OM) during elemental analysis (EA), IRMS and AMS. S1 text details expected relationships between the amount of plastic contamination in organic matter samples and the following measurable quantities.

1. The mass-yields ($y_{\Sigma}$) and molar yields ($n_{\Sigma}$) of carbon (C) and nitrogen (N)
2. The mass-proportions ($\hat{p}$) of carbon (subscript C) and nitrogen (subscript N) where, for example, $\hat{p}_{C}$= mass of carbon per mass of substance and thus $0\leq\hat{p}\leq1$. However, $\hat{p}$ can be expressed as a “weight-percent” after multiplying by a factor of 100 %.
3. C:N ratios
4. Carbon isotopic abundances (δ^13^C and Δ^14^C) and radiocarbon ages ($t$).

Key equations are highlighted in gray.

# Text S1

# Carbon

## Combustion efficiency

During elemental analysis (EA), small volumes of solid organic matter are flash combusted to CO_2_ in an atmosphere of excess oxygen. The reactions can be expressed in terms of the carbon atoms in natural organic matter (organic carbon, OC) and plastic (PC), assuming that the total abundances of all oxidation byproducts besides CO_2_ are negligible (e.g., CO).

| $\text{OC}+\text{O}_{2}\to\text{CO}_{2}$ | (S1) |
| --- | --- |
|  |  |
| $\text{PC}+\text{O}_{2}\to\text{CO}_{2}$ | (S2) |

The residual molar abundances of OC and PC can be parameterized in terms of their initial abundances (subscript *o*) and the number of forward reactions that have taken place (the “extent of reaction”, $\xi$).

| $\text{OC}=\text{OC}_{o}-\xi_{\text{OC}}$ | (S3) |
| --- | --- |
|  |  |
| $\text{PC}=\text{PC}_{o}-\xi_{\text{PC}}$ | (S4) |

The stoichiometries of Eq. (S1 – S2) dictate that one mole of CO_2_ is produced for each mole of either OC or PC that is combusted. Therefore, the amount of CO_2_ produced during combustion is equal to the extent of reaction (e.g., $\xi_{\text{PC}}=\text{PC}_{o}-\text{PC}=\text{CO}_{2}$). Likewise, the maximum extent of reaction, and hence the maximum amount of CO_2_ produced, will occur when the reactant is completely consumed (e.g., $\xi_{\text{PC,max}}=\text{PC}_{o}=\text{CO}_{2}$). Therefore, the efficiency of either reaction (Eq. (S1) or (S2)) is equal to the ratio of the final- to maximum possible extents of reaction ($\epsilon=\xi_{final}/\xi_{\text{max}}$), and can be calculated from two measurable quantities: the amounts of CO_2_ produced and the initial amounts of carbon in the reactants.

| $\epsilon_{\text{OC}}= \frac{\text{CO}_{2} \text{from OC}}{\text{OC}_{o}}$ | (S5) |
| --- | --- |
|  |  |
| $\epsilon_{\text{PC}}=\frac{\xi_{\text{PC}}}{\text{PC}_{o}}=\frac{\text{CO}_{2} \text{from PC}}{\text{PC}_{o}}$ | (S6) |

## Conservation of mass

The total mass ($m_{\Sigma}$) of plastic (subscript p) and organic matter (subscript OM) in a mixture before combustion is equal to the sum of their respective masses.

| $m_{\Sigma}=m_{\text{om}}+m_{\text{p}}$ | (S7) |
| --- | --- |

Likewise, the total mass of carbon atoms ($m_{\Sigma\text{C}}$) in that mixture prior to combustion is the sum of the masses of carbon from each component, which are equal to the products of their respective masses and mass-proportions of carbon ($\hat{p}_{\text{oc}}$ and $\hat{p}_{\text{pc}}$).

| $m_{\Sigma\text{C}}=\hat{p}_{\Sigma\text{C}}m_{\Sigma}=\hat{p}_{\text{oc}}m_{\text{om}}+\hat{p}_{\text{pc}}m_{\text{p}}$ | (S8) |
| --- | --- |

The mass yield of carbon ($y_{\Sigma,\text{C}}$), expressed as the mass of C in CO_2_ produced during combustion, will depend on the combustion efficiencies of each component (Eq. (S5 – S6)). It will be assumed hereafter that the efficiencies are constant across all attributes of either component, e.g., regardless of molecular structure or particle size.

| $y_{\Sigma,\text{C}}=\epsilon_{\Sigma C}\hat{p}_{\Sigma\text{C}}m_{\Sigma}=\epsilon_{\text{oc}}\hat{p}_{\text{oc}}m_{\text{om}}+\epsilon_{\text{pc}}\hat{p}_{\text{pc}}m_{\text{p}}$ | (S9) |
| --- | --- |

Each term in this equation represents a mass of carbon atoms. If these materials have natural carbon isotopic abundances (~99 % ^12^C and ~1 % ^13^C) and if isotopic fractionation during combustion is negligible, then the moles of carbon atoms ($n=n_{{}^{12}C}+n_{{}^{13}C}$) in the CO_2_ produced during combustion can be estimated by dividing Eq. (S9) by the molar mass of carbon ($mw_{\text{C}}$ = 12.011 g mol^-1^).

| $n_{\Sigma,\text{C}}=\epsilon_{\Sigma C}\frac{\hat{p}_{\Sigma\text{C}}m_{\Sigma}}{mw_{\text{C}}}=\epsilon_{\text{oc}}\frac{\hat{p}_{\text{oc}}m_{\text{om}}}{mw_{\text{C}}}+\epsilon_{\text{pc}}\frac{\hat{p}_{\text{pc}}m_{\text{p}}}{mw_{\text{C}}}$ | (S10) |
| --- | --- |

Eq. (S9) and (S10) are basic statements of conservation of mass from which the following important theoretical conclusions can be drawn about EA analyses of organic matter that is contaminated with plastic.

## Expected yield of carbon

If the combustion efficiencies of plastic carbon and organic carbon approach 1 (i.e., 100 % combustion), then the yields (Eq. (S9) and (S10)) will simplify to Eq. (S11) and (S12). If the measured yields follow these relationships, then it could be assumed that plastic contamination is quantitatively co-combusted with natural organic matter.

|  |  |
| --- | --- |
| $y_{\Sigma,\text{C}}=\hat{p}_{\Sigma\text{C}}m_{\Sigma}=\hat{p}_{\text{oc}}m_{\text{om}}+\hat{p}_{\text{pc}}m_{\text{p}}$ | (S11) |
|  |  |
| $n_{\Sigma,\text{C}}=\frac{\hat{p}_{\Sigma\text{C}}m_{\Sigma}}{mw_{\text{C}}}=\frac{\hat{p}_{\text{oc}}m_{\text{om}}}{mw_{\text{C}}}+\frac{\hat{p}_{\text{pc}}m_{\text{p}}}{mw_{\text{C}}}$ | (S12) |
|  |  |

## Expected mass-proportion of carbon

Based on Eq. (S9) or (S10), the mass proportion of carbon in the mixture is equal to the yield divided by the total mass of the mixture ($m_{\Sigma}$) and its total combustion efficiency ($\epsilon_{\Sigma}$).

| $\hat{p}_{\Sigma\text{C}}=\frac{\epsilon_{\text{oc}}}{\epsilon_{\Sigma C}}\hat{p}_{\text{oc}}\left( \frac{m_{\text{om}}}{m_{\Sigma}} \right)+\frac{\epsilon_{\text{pc}}}{\epsilon_{\Sigma C}}\hat{p}_{\text{pc}}\left( \frac{m_{\text{p}}}{m_{\Sigma}} \right)$ | (S13) |
| --- | --- |

The terms in parentheses are *mass* fractions: the fractions of the total masses (i.e., not solely of carbon) of organic matter or plastic in the original mixture:

| $x_{\text{p}}=\frac{m_{\text{p}}}{m_{\Sigma}}$ | (S14) |
| --- | --- |
|  |  |
| $x_{\text{om}}=\frac{m_{\text{om}}}{m_{\Sigma}}$ | (S15) |

The mass fractions of organic matter and plastic must add to one ($x_{\text{p}}+x_{\text{om}}=1$), assuming they are the only materials in the mixture. Therefore, Eq. (S13) can be rewritten in terms of either mass fraction by substitution:

| $\hat{p}_{\Sigma\text{C}}=\left( \frac{\epsilon_{\text{pc}}}{\epsilon_{\Sigma C}}\hat{p}_{\text{pc}}-\frac{\epsilon_{\text{oc}}}{\epsilon_{\Sigma C}}\hat{p}_{\text{oc}} \right)x_{\text{p}}+\frac{\epsilon_{\text{oc}}}{\epsilon_{\Sigma C}}\hat{p}_{\text{oc}}$ | (S16) |
| --- | --- |
|  |  |
| $\hat{p}_{\Sigma\text{C}}=\left( \frac{\epsilon_{\text{oc}}}{\epsilon_{\Sigma C}}\hat{p}_{\text{oc}}-\frac{\epsilon_{\text{pc}}}{\epsilon_{\Sigma C}}\hat{p}_{\text{pc}} \right)x_{\text{om}}+\frac{\epsilon_{\text{oc}}}{\epsilon_{\Sigma C}}\hat{p}_{\text{pc}}$ | (S17) |

Assuming Eq. (S1) and (S2) are the only reactions taking place, that plastic and organic carbon are combusted independently, and that their combustion efficiencies approach 1, then Eq. (S16) and (S17) simplify to Eq. (S18) and (S19).

|  |  |
| --- | --- |
| $\hat{p}_{\Sigma\text{C}}=\left( \hat{p}_{\text{pc}}-\hat{p}_{\text{oc}} \right)x_{\text{p}}+\hat{p}_{\text{oc}}$ | (S18) |
|  |  |
| $\hat{p}_{\Sigma\text{C}}=\left( \hat{p}_{\text{oc}}-\hat{p}_{\text{pc}} \right)x_{\text{om}}+\hat{p}_{\text{pc}}$ | (S19) |
|  |  |

If the measured mass-proportions of CO_2_ follow these relationships, then the EA analyses are consistent with plastic contamination being quantitatively co-combusted with natural organic matter.

The percent carbon can also be expressed as a function of the *mole* fractions (capital $X$) of carbon: the fractions of all carbon atoms in the mixture ($n_{\Sigma\text{C}}$) derived from plastic ($X_{\text{pc}}={n_{\text{pc}}}/{n_{\Sigma\text{C}}}$) or organic matter ($X_{\text{oc}}={n_{\text{oc}}}/{n_{\Sigma\text{C}}}$). These ratios can be calculated from the corresponding masses and mass proportions of carbon, and are thus directly proportional to the mass fractions of each component:

| $X_{\text{pc}}=\frac{\hat{p}_{\text{pc}}m_{\text{p}}}{\hat{p}_{\text{ΣC}}m_{\Sigma}}=\frac{\hat{p}_{\text{pc}}}{\hat{p}_{\text{Σ}}}x_{\text{p}}$ | (S20) |
| --- | --- |
|  |  |
| $X_{\text{oc}}=\frac{\hat{p}_{\text{oc}}m_{\text{om}}}{\hat{p}_{\text{ΣC}}m_{\Sigma}}=\frac{\hat{p}_{\text{oc}}}{\hat{p}_{\text{ΣC}}}x_{\text{om}}$ | (S21) |

Substituting Eq. (S20) and (S21) into Eq. (S18) and (S19) and noting that $X_{\text{pc}}+X_{\text{oc}}=1$, reveals the mass-proportion of carbon in a mixture as a hyperbolic function of the mole fraction of carbon originating from either plastic or organic matter.

| $\frac{1}{\hat{p}_{\Sigma\text{C}}}=\frac{1}{\hat{p}_{\text{oc}}}+\left( \frac{1}{\hat{p}_{\text{pc}}}-\frac{1}{\hat{p}_{\text{oc}}} \right)X_{\text{pc}}$ | (S22) |
| --- | --- |
|  |  |
| $\frac{1}{\hat{p}_{\Sigma\text{C}}}=\frac{1}{\hat{p}_{\text{pc}}}+\left( \frac{1}{\hat{p}_{\text{oc}}}-\frac{1}{\hat{p}_{\text{pc}}} \right)X_{\text{oc}}$ | (S23) |

Eq. (S22) and (S23), combined with analogous expressions for the anticipated mass-proportions of nitrogen, are useful for deriving the anticipated relationship between C:N ratios and the proportion of plastic contamination in a sample of organic matter (below).

# Nitrogen

The expected mass yields ($y_{\Sigma\text{N}}$), molar yields ($n_{\Sigma\text{N}}$), and mass-proportions of nitrogen ($\hat{p}_{\Sigma\text{N}}$) in mixtures of plastic and organic matter can be found by following the same logic and assumptions as outlined above for carbon.

## Expected yield of nitrogen

The expected mass yield of nitrogen ($y_{\Sigma\text{N}}$) assuming 100 % combustion is the sum of the masses of nitrogen originating from organic matter and plastic, which are equal to the products of their respective total masses ($m_{\text{om}}$ and $m_{\text{p}}$) and mass-proportions of nitrogen ($\hat{p}_{\text{o}\text{N}}$ and $\hat{p}_{\text{p}\text{N}}$) (Eq. (S24)). The expected molar yield of nitrogen ($n_{\Sigma\text{N}}$) can be found by dividing the expected mass yield ($y_{\Sigma\text{N}}$) by the molar mass of nitrogen ($mw_{\text{N}}=14.007 \text{g }\text{mol}^{-1}$), assuming the organic matter and plastic nitrogen have natural abundance ^15^N contents (~0.37 % ^15^N).

| $y_{\Sigma N}=\hat{p}_{\Sigma N}m_{\Sigma}=\hat{p}_{\text{o}\text{N}}m_{\text{om}}+\hat{p}_{\text{p}\text{N}}m_{\text{p}}$ | (S24) |
| --- | --- |
|  |  |
| $n_{\Sigma,N}=\frac{\hat{p}_{\Sigma N}m_{\Sigma}}{mw_{N}}=\frac{\hat{p}_{\text{o}\text{N}}m_{\text{om}}}{mw_{N}}+\frac{\hat{p}_{\text{p}\text{N}}m_{\text{p}}}{mw_{N}}$ | (S25) |

These equations simplify to Eq. (S26) and (S27) for plastics devoid of nitrogen ($\hat{p}_{\text{PN}}\to0$).

|  |  |
| --- | --- |
| $y_{\Sigma N}=\hat{p}_{\Sigma N}m_{\Sigma}=\hat{p}_{\text{o}\text{N}}m_{\text{om}}$ | (S26) |
|  |  |
| $n_{\Sigma,\text{C}}=\frac{\hat{p}_{\Sigma N}m_{\Sigma}}{mw_{N}}=\frac{\hat{p}_{\text{o}\text{N}}m_{\text{om}}}{mw_{N}}$ | (S27) |
|  |  |

In such cases, the yields of nitrogen ($y_{\Sigma N}$ and $n_{\Sigma N}$) would be independent of the mass fraction of plastic in the mixture, but plastic will still contribute to the total mass of the mixture. Therefore, contamination with nitrogen-free plastics would artifactually decrease the total mass-proportion of nitrogen ($\hat{p}_{\Sigma N}$) in all contaminated samples, except in fortuitous cases where $\hat{p}_{\text{o}\text{N}}=\hat{p}_{\text{PN}}$.

## Expected mass-proportion of nitrogen

As for carbon, the measured mass proportions of nitrogen in fully combusted mixtures should be linear functions of the mass fractions of plastic and organic matter.

| $\hat{p}_{\Sigma N}=\left( \hat{p}_{\text{p}\text{N}}-\hat{p}_{\text{o}\text{N}} \right)x_{\text{p}}+\hat{p}_{\text{o}\text{N}}$ | (S28) |
| --- | --- |
|  |  |
| $\hat{p}_{\Sigma N}=\left( \hat{p}_{\text{o}\text{N}}-\hat{p}_{\text{p}\text{N}} \right)x_{\text{om}}+\hat{p}_{\text{p}\text{N}}$ | (S29) |

As for carbon, we can define the mole fractions of nitrogen originating from plastic ($X_{\text{PN}}$) and organic matter ($X_{\text{ON}}$).

| $X_{\text{PN}}=\frac{\hat{p}_{\text{p}\text{N}}m_{\text{p}}}{\hat{p}_{\text{ΣN}}m_{\Sigma}}=\frac{\hat{p}_{\text{p}\text{N}}}{\hat{p}_{\text{ΣN}}}x_{\text{p}}$ | (S30) |
| --- | --- |
|  |  |
| $X_{\text{o}\text{N}}=\frac{\hat{p}_{\text{o}\text{N}}m_{\text{om}}}{\hat{p}_{\text{ΣN}}m_{\Sigma}}=\frac{\hat{p}_{\text{o}\text{N}}}{\hat{p}_{\text{ΣN}}}x_{\text{om}}$ | (S31) |

Solving these definitions for the mass fractions of plastic and organic matter, substituting into Eq. (S28 – S29), and simplifying predicts that mass proportion of nitrogen will be a hyperbolic function of the mole fraction of nitrogen originating from plastic or organic matter.

| $\frac{1}{\hat{p}_{\Sigma N}}=\frac{1}{\hat{p}_{\text{o}\text{N}}}+\left( \frac{1}{\hat{p}_{\text{p}\text{N}}}-\frac{1}{\hat{p}_{\text{o}\text{N}}} \right)X_{\text{p}\text{N}}$ | (S32) |
| --- | --- |
|  |  |
| $\frac{1}{\hat{p}_{\Sigma N}}=\frac{1}{\hat{p}_{\text{p}\text{N}}}+\left( \frac{1}{\hat{p}_{\text{o}\text{N}}}-\frac{1}{\hat{p}_{\text{p}\text{N}}} \right)X_{\text{o}\text{N}}$ | (S33) |

All expressions for the mass-proportion of nitrogen (Eq. (S28), (S29), (S32), (S33)) are simpler for plastics devoid of nitrogen ($\hat{p}_{\text{p}\text{N}}\to0$).

|  |  |
| --- | --- |
| $\hat{p}_{\Sigma N}=\hat{p}_{\text{o}\text{N}}-\hat{p}_{\text{o}\text{N}}x_{\text{p}}$ | (S34) |
|  |  |
| $\hat{p}_{\Sigma N}=\hat{p}_{\text{o}\text{N}}x_{\text{om}}$ | (S35) |
|  |  |
| $\hat{p}_{\Sigma N}=\frac{\hat{p}_{\text{o}\text{N}}}{1-X_{\text{p}\text{N}}}$ | (S36) |
|  |  |
| $\hat{p}_{\Sigma N}=\frac{\hat{p}_{\text{o}\text{N}}}{X_{\text{o}\text{N}}}$ | (S37) |
|  |  |

# Expected C:N Ratio

The C:N ratio in a mixture ($\text{C:N}_{\Sigma}$) of plastic and organic is the ratio the total moles of carbon atoms ($n_{\Sigma C}$) to nitrogen atoms ($n_{\Sigma N}$) atoms:

| $\text{C:N}_{\Sigma}=\frac{n_{\Sigma\text{C}}}{n_{\Sigma N}}$ | (S38) |
| --- | --- |

Substituting Eq. (S12) for $n_{\Sigma\text{C}}$, Eq. (S25) for $n_{\Sigma N}$, applying the definitions for mass fractions of plastic and organic matter (Eq. (S14) and (S15)), and simplifying reveals that the expected $\text{C:N}_{\Sigma}$ ratio is a hyperbolic function of the mass fraction of plastic ($x_{\text{p}}$) in the mixture.

| $\text{C:N}_{\Sigma}=\frac{mw_{\text{N}}}{mw_{\text{C}}}\left( \frac{\left( \hat{p}_{\text{pc}}-\hat{p}_{\text{oc}} \right)x_{\text{p}}+\hat{p}_{\text{oc}}}{\left( \hat{p}_{\text{p}\text{N}}-\hat{p}_{\text{o}\text{N}} \right)x_{\text{p}}+\hat{p}_{\text{o}\text{N}}} \right)$ | (S39) |
| --- | --- |

The $\text{C:N}_{\Sigma}$ ratio fundamentally depends on the number of carbon atoms in the mixture and therefore is more conveniently expressed as a function of the mole fraction of carbon from plastic. This relationship can be found quickly by noting that the numerator in parentheses of Eq. (S39) is simply the total mass-proportion of C in the mixture ($\hat{p}_{\Sigma\text{C}}$, i.e., Eq. (S18)). Applying the hyperbolic relationship between $\hat{p}_{\Sigma\text{C}}$ and $X_{\text{pc}}$ (Eq. (S22)), substituting the mole fraction of plastic carbon for mass fraction of plastic (Eq. (S20)) in the denominator of Eq. (S39), and simplifying produces the desired relationship:

| $\text{C:N}_{\Sigma}=\frac{\left( \frac{mw_{\text{N}}}{mw_{\text{C}}} \right)}{\left( \frac{\hat{p}_{\text{pN}}}{\hat{p}_{\text{pc}}}-\frac{\hat{p}_{\text{o}\text{N}}}{\hat{p}_{\text{oc}}} \right)X_{\text{pc}}+\frac{\hat{p}_{\text{o}\text{N}}}{\hat{p}_{\text{oc}}}}$ | (S40) |
| --- | --- |

If there is no plastic in the mixture ($X_{\text{pc}}=0$), then this equation reduces to the $\text{C:N}$ ratio of pure organic matter ($\text{C:N}_{\mathrm{om}}$).

| $\text{C:N}_{\Sigma}=\left( \frac{mw_{\text{N}}}{mw_{\text{C}}} \right)\frac{\hat{p}_{\text{oc}}}{\hat{p}_{\text{o}\text{N}}}=\text{C:N}_{\mathrm{om}}$ | (S41) |
| --- | --- |

If there is no organic matter in the mixture ($X_{\text{pc}}=1$), then this equation reduces to the $\text{C:N}$ ratio of pure plastic ($\text{C:N}_{p}$).

| $\text{C:N}_{\Sigma}=\left( \frac{mw_{\text{N}}}{mw_{\text{C}}} \right)\frac{\hat{p}_{\text{pc}}}{\hat{p}_{\text{pN}}}=\text{C:N}_{\text{p}}$ | (S42) |
| --- | --- |

Based on these definitions (Eq. (S41) and (S42)), the $\text{C:N}_{\Sigma}$ ratio is a hyperbolic function of the $\text{C:N}$ ratios of pure plastic and organic matter, and of the mole fraction of plastic carbon contamination:

|  |  |
| --- | --- |
| $\text{C:N}_{\Sigma}=\frac{\text{C:N}_{\text{om}} \text{C:N}_{\text{p}}}{\left( \text{C:N}_{\text{om}}-\text{C:N}_{\text{p}} \right)X_{\text{pc}}+\text{C:N}_{\text{p}}}$  Or, equivalently:  $\frac{1}{\text{C:N}_{\Sigma}}=\left( \frac{1}{\text{C:N}_{\text{p}}}-\frac{1}{\text{C:N}_{\text{om}}} \right)X_{\text{pc}}+\frac{1}{\text{C:N}_{\text{om}}}$ | (S43) |
|  |  |

These relationships are simpler for plastics devoid of nitrogen ($\text{C:N}_{\text{p}}\to\infty$), such as polystyrene.

|  |  |
| --- | --- |
| $\text{C:N}_{\Sigma}=\frac{\text{C:N}_{\text{om}}}{1-X_{\text{pc}}}$  Or, equivalently:  $\frac{1}{\text{C:N}_{\Sigma}}=\frac{1}{\text{C:N}_{\text{om}}}-\frac{1}{\text{C:N}_{\text{om}}}X_{\text{pc}}$ | (S44) |
|  |  |

**The relationship between mass fraction of plastic and mole fraction of plastic carbon atoms**

The relationship between the mass fraction of plastic and the mole fraction of plastic carbon atoms can be found by conservation of mass and the following definitions.

The proportion of carbon by mass in pure plastic ($\hat{p}_{\text{pc}}$) is defined as the ratio of the mass of plastic carbon ($m_{\text{pc}}$) to the total mass of plastic ($m_{\text{p}}$). These proportions range from 0 to 1 and can be expressed as a percentage after multiplying by 100 (i.e., $\%C=\hat{p}_{\text{pc}}\times100 \%$). The proportion of carbon by mass in organic matter ($\hat{p}_{\text{oc}}$) is similarly defined:

|  |  |
| --- | --- |
| $\hat{p}_{\text{pc}}=\frac{m_{\text{pc}}}{m_{\text{p}}}$ | (S45) |
|  |  |
| $\hat{p}_{\text{oc}}=\frac{m_{\text{oc}}}{m_{\text{om}}}$ | (S46) |
|  |  |

The mass fractionsof plastic ($x_{\text{p}}$) or of organic matter ($x_{\text{om}}$) in a mixture are defined as the ratios of the masses of plastic ($m_{\text{p}}$), or organic matter ($m_{\text{om}}$), to the total mass of the mixture ($m_{\Sigma}=m_{\text{om}}+m_{\text{p}}$), respectively.

|  |  |
| --- | --- |
| $x_{\text{p}}=\frac{m_{\text{p}}}{m_{\Sigma}}$ | (S47) |
|  |  |
| $x_{\text{om}}=\frac{m_{\text{om}}}{m_{\Sigma}}$ | (S48) |
|  |  |

Conservation of mass dictates that the sum of the mass fractions must be equal to 1 ($x_{\text{p}}+x_{\text{om}}=1$). Therefore, the proportion of carbon in a mixture of plastic and organic matter is equal to the sum of their individual proportions of carbon weighted by their respective mass fractions in the mixture. This relationship can be simplified to linear functions of the mass fractions of either plastic or organic matter in the mixture (Medina Faull, 2022).

|  |  |
| --- | --- |
| $\hat{p}_{\text{ΣC}}=\left( \hat{p}_{\text{pc}}-\hat{p}_{\text{oc}} \right)x_{\text{p}}+\hat{p}_{\text{oc}}$ | (S49) |
|  |  |
| $\hat{p}_{\text{ΣC}}=\left( \hat{p}_{\text{oc}}-\hat{p}_{\text{pc}} \right)x_{\text{om}}+\hat{p}_{\text{pc}}$ | (S50) |
|  |  |

The ratio of the number of plastic carbon atoms ($n_{\text{pc}}$) to the number of all carbon atoms ($n_{\Sigma c}$) in the mixture is the mole fraction of plastic carbon ($X_{\text{pc}}$). A mole fraction of organic carbon atoms is defined in a similar manner:

|  |  |
| --- | --- |
| $X_{\text{pc}}=\frac{n_{\text{pc}}}{n_{\Sigma\text{c}}}$ | (S51) |
|  |  |
| $X_{\text{oc}}=\frac{n_{\text{oc}}}{n_{\Sigma\text{c}}}$ | (S52) |
|  |  |

The number of moles of carbon is equal to the mass of a material (e.g., $m_{\text{p}}$) times its proportion of carbon by mass (e.g., $\hat{p}_{\text{pc}}$) and divided by carbon’s atomic mass (12.011 g mol^-1^). Therefore, Eq. (S51) and (S52) can be written in terms of the proportions of carbon in plastic, organic matter, and their mixtures, and the mole fraction of carbon from plastic or organic matter. Carbon’s atomic mass cancels from the numerator and denominator leading to the following equations.

|  |  |
| --- | --- |
| $X_{\text{pc}}=\frac{\hat{p}_{\text{pc}}m_{\text{p}}}{\hat{p}_{\text{ΣC}}m_{\text{Σ}}}=\frac{\hat{p}_{\text{pc}}}{\hat{p}_{\text{ΣC}}}x_{\text{pc}}$ | (S53) |
|  |  |
| $X_{\text{oc}}=\frac{\hat{p}_{\text{oc}}m_{\text{om}}}{\hat{p}_{\text{ΣC}}m_{\text{ΣC}}}=\frac{\hat{p}_{\text{oc}}}{\hat{p}_{\text{ΣC}}}x_{\text{oc}}$ | (S54) |
|  |  |

Substituting Eq. (S49) for $\hat{p}_{\text{ΣC}}$ in Eq. (S52) and simplifying gives the relationship between the mass fraction and mole fraction of plastic carbon in a mixture with organic matter.

|  |  |
| --- | --- |
| $X_{\text{pc}}=\frac{1}{1+\left( \frac{1}{x_{\text{p}}}-1 \right)\frac{\hat{p}_{\text{oc}}}{\hat{p}_{\text{pc}}}}$ | (S55) |
| Or, equivalently: |  |
| $\frac{1}{X_{\text{pc}}}=1+\left( \frac{1}{x_{\text{p}}}-1 \right)\frac{\hat{p}_{\text{oc}}}{\hat{p}_{\text{pc}}}$ | (S56) |
|  |  |

Solving for $x_{\text{p}}$ gives the mass fraction as a function of the mole fraction carbon in the mixture and of the proportions of carbon in each endmember.

|  |  |
| --- | --- |
| $x_{\text{pc}}=\frac{1}{1+\left( \frac{1}{X_{\text{p}}}-1 \right)\frac{\hat{p}_{\text{pc}}}{\hat{p}_{\text{oc}}}}$ | (S57) |
| Or, equivalently: |  |
| $\frac{1}{x_{\text{p}}}=1+\left( \frac{1}{X_{\text{pc}}}-1 \right)\frac{\hat{p}_{\text{pc}}}{\hat{p}_{\text{oc}}}$ | (S58) |
|  |  |

Note that analogous relationships can be derived for the nitrogen composition by replacing all “c” subscripts with “N”, i.e., $\hat{p}_{\text{pN}}$, $\hat{p}_{\text{oN}}$, $\hat{p}_{\text{ΣN}}$, $n_{\text{pN}}$, $n_{\text{oN}}$, $X_{\text{pN}}$, $X_{\text{oN}}$.

# Expected Isotopic Compositions

The terms in Eq. (S12) represent the number of moles of carbon atoms derived from organic matter, from plastic, or in their ensuing mixture, assuming 100 % combustion and that all materials have near natural fractional isotopic abundances of ^13^C (i.e., ${}^{13}f={n_{{}^{13}C}}/\left( n_{{}^{12}C}+n_{{}^{13}C} \right)$ ~ 1 % and $mw_{\text{C}}=12.011 \text{g }\text{mol}^{-1}$). Therefore, multiplying each term in Eq. (S12) by its associated fractional abundance and simplifying produces an independent equation for the conservation of mass of ^13^C atoms.

| ${{}^{13}f}_{\Sigma}\hat{p}_{\Sigma\text{C}}m_{\Sigma}={{}^{13}f}_{\text{oc}}\hat{p}_{\text{oc}}m_{\text{om}}+{{}^{13}f}_{\text{pc}}\hat{p}_{\text{pc}}m_{\text{p}}$ | (S59) |
| --- | --- |

Dividing Eq. (S59) by the total mass of carbon ($\hat{p}_{\Sigma\text{C}}m_{\Sigma}$) and applying the definitions of mole fractions (Eq. (S20) and (S21)) to the ensuing quotients produces the canonical relationship for the isotopic composition of two component mixtures, expressed here in terms of the mole fractions of plastic or organic carbon.

| ${{}^{13}f}_{\Sigma}=\left( {{{}^{13}f}_{\text{pc}}-{}^{13}f}_{\text{oc}} \right)X_{\text{pc}}+{{}^{13}f}_{\text{oc}}$ | (S60) |
| --- | --- |
|  |  |
| ${{}^{13}f}_{\Sigma}=\left( {{}^{13}f}_{\text{oc}}-{{}^{13}f}_{\text{pc}} \right)X_{\text{oc}}+{{}^{13}f}_{\text{pc}}$ | (S61) |

These equations are exact isotopic expressions for conservation of mass with respect to ^12^C and ^13^C atoms under the assumptions stated, above. Analogous isotopic expressions for conservation of nitrogen atoms could be derived using fractional isotopic abundances of ^15^N and applied to mixtures with plastics that contain nitrogen.

## δ^13^C values and errors

For materials with natural ^13^C abundances (${}^{13}f\sim1 \%)$, Eq. (S60 – S61) can be approximated with negligible error by substituting δ^13^C values for fractional isotopic abundances.

| ${\delta{}^{13}C}_{\Sigma}=\left( \delta{{}^{13}C}_{\text{pc}}-{\delta{}^{13}C}_{\text{oc}} \right)X_{\text{pc}}+\delta{{}^{13}C}_{\text{oc}}$ | (S62) |
| --- | --- |
|  |  |
| $\delta{{}^{13}C}_{\Sigma}=\left( {\delta{}^{13}C}_{\text{oc}}-\delta{{}^{13}C}_{\text{pc}} \right)X_{\text{oc}}+\delta{{}^{13}C}_{\text{pc}}$ | (S63) |

The error in a measured δ^13^C value is the difference between the measured and actual δ^13^C value of organic carbon in a sample contaminated with plastic: δ^13^C error = ${\delta{}^{13}C}_{\Sigma}-\delta{{}^{13}C}_{\text{oc}}$. Based on Eq. (S62), this error is directly proportional to the mole fraction of plastic carbon contamination, and to the difference between the δ^13^C signatures of the organic and plastic carbon.

|  |  |
| --- | --- |
| $\delta{}^{13}C \text{error}=\left( \delta{{}^{13}C}_{\text{pc}}-{\delta{}^{13}C}_{\text{oc}} \right)X_{\text{pc}}$ | (S64) |
|  |  |

## Radiocarbon Δ^14^C values and errors

Equations analogous to those for δ^13^C can be derived for the expected radiocarbon (^14^C) abundance of an organic matter sample contaminated with plastic using Δ^14^C values, assuming both materials have natural ^14^C abundances (≤  *ca.* 10^-12 14^C/^12^C atoms).

|  |  |
| --- | --- |
| ${\Delta{}^{14}C}_{\Sigma}=\left( \Delta{{}^{14}C}_{\text{pc}}-{\Delta{}^{14}C}_{\text{oc}} \right)X_{\text{pc}}+\Delta{{}^{14}C}_{\text{oc}}$ | (S65) |
|  |  |
| ${\Delta{}^{14}C}_{\Sigma}=\left( \Delta{{}^{14}C}_{\text{oc}}-{\Delta{}^{14}C}_{\text{pc}} \right)X_{\text{oc}}+\Delta{{}^{14}C}_{\text{pc}}$ | (S66) |
|  |  |
| ${\Delta{}^{14}C}_{\Sigma} \text{error}=\left( \Delta{{}^{14}C}_{\text{pc}}-{\Delta{}^{14}C}_{\text{oc}} \right)X_{\text{pc}}$ | (S67) |
|  |  |

Eq. (S65) – (S67) are simpler for plastics manufactured from fossil fuels, which are millions of years old and devoid of ^14^C atoms ($\Delta{{}^{14}C}_{\text{pc}}=-1000 ‰$ by definition).

|  |  |
| --- | --- |
| ${\Delta{}^{14}C}_{\Sigma}=\left( -1000-{\Delta{}^{14}C}_{\text{oc}} \right)X_{\text{pc}}+\Delta{{}^{14}C}_{\text{oc}}$ | (S68) |
|  |  |
| ${\Delta{}^{14}C}_{\Sigma}=\left( \Delta{{}^{14}C}_{\text{oc}}+1000 \right)X_{\text{oc}}-1000$ | (S69) |
|  |  |
| ${\Delta{}^{14}C}_{\Sigma} \text{error}=-\left( 1000+{\Delta{}^{14}C}_{\text{oc}} \right)X_{\text{pc}}$ | (S70) |
|  |  |

## Radiocarbon ages and errors

Conventional ^14^C ages ($t$) can be calculated from Δ^14^C values based on the definition below (Eq. (S71)), where 5568 is the “Libby” ^14^C half-life, 5730 y is the “Cambridge” ^14^C half-life, 1950 is the reference year used to report all conventional ^14^C ages, and “y” is the relevant year of interest for a given sample, e.g., the year it was collected, measured, grown, or deposited.

| $t=-\frac{5568}{\ln\left( 2 \right)}\ln\left( \frac{\Delta{}^{14}C}{1000}+1 \right)+\frac{5568}{5730}\left( 1950-y \right)$ | (S71) |
| --- | --- |

Solving Eq. (S71) for Δ^14^C values as functions of the conventional ^14^C ages of plastic ($t_{p}$), organic carbon ($t_{oc}$), and their mixture ($t_{\Sigma}$), then substituting into Eq. (S65) and assuming the year of interest ($y$) is the same for plastic and organic carbon, reveals the relationship between the conventional ^14^C ages of a contaminated samples and its components.

| $e^{-\left( \frac{t_{\Sigma}\ln\left( 2 \right)}{5568} \right)}=\left[ e^{-\left( \frac{t_{\text{p}}\ln\left( 2 \right)}{5568} \right)}-e^{-\left( \frac{t_{\text{oc}}\ln\left( 2 \right)}{5568} \right)} \right]X_{\text{pc}}+e^{-\left( \frac{t_{\text{oc}}\ln\left( 2 \right)}{5568} \right)}$ | (S72) |
| --- | --- |

Multiplying Eq. (S72) by a factor of $e^{\left( \frac{t_{\text{oc}}\ln\left( 2 \right)}{5568} \right)}$, computing the natural logarithm of the ensuing equation, and simplifying leads to an expression for the age error ($t \text{error}=t_{\Sigma}-t_{\text{oc}}$) as a function of the mole fraction of plastic carbon contamination.

|  |  |
| --- | --- |
| $t \text{error}=-\frac{5568}{\ln\left( 2 \right)}\ln\left\{ 1+\left[ e^{-\left( \frac{\left( t_{\text{p}}-t_{\text{oc}} \right)\ln\left( 2 \right)}{5568} \right)}-1 \right]X_{\text{pc}} \right\}$ | (S73) |
|  |  |

The exponential term approaches zero for plastics manufactured from fossil fuel, because their theoretical conventional ^14^C ages (millions of years) are orders of magnitude larger than those of either organic matter from the sea or the Libby ^14^C half-life. In such cases, Eq. (S73) reduces to the following much simpler function of the mole fraction of plastic carbon contamination:

|  |  |
| --- | --- |
| $t \text{error ≈}-\frac{5568}{\ln\left( 2 \right)}\ln\left( 1-X_{\text{pc}} \right)$ | (S74) |
|  |  |

By Taylor-series approximation, $\ln\left( 1-X_{\text{pc}} \right)\approx-X_{\text{pc}}$ for slightly contaminated samples (i.e., for $X_{\text{pc}}\leq0.1$, or equivalently, less than 10 mole percent plastic carbon). In such cases, $t \text{error ≈ 8033 }X_{\text{pc}}$, which represent an overestimate of ~80 ^14^C-years for each mole percent of plastic carbon contamination.

**The relationship between δ^13^C and C:N in mixtures of MP and natural organic matter**

Based on conservation of mass, the total C:N ratio in a mixture (${C:N}_{\Sigma}$) of plastic and natural organic matter can be calculated from the C:N ratios of these endmembers (${C:N}_{\text{p}}$ and ${C:N}_{\text{om}}$) and the mole fraction of plastic carbon atoms in the mixture ($X_{\text{pc}}$) (Medina Faull, 2022).

|  |  |
| --- | --- |
| $\frac{1}{{C:N}_{\Sigma}}=\left( \frac{1}{{C:N}_{\text{p}}}-\frac{1}{{C:N}_{\text{om}}} \right)X_{\text{pc}}+\frac{1}{{C:N}_{\text{om}}}$ | (S75) |
|  |  |

Likewise, the δ^13^C value of the mixture is a linear function of the δ^13^C values of each endmember and the mole fraction of plastic in the mixture:

|  |  |
| --- | --- |
| $\delta{{}^{13}C}_{\Sigma}=\left( \delta{{}^{13}C}_{\text{pc}}-\delta{{}^{13}C}_{\text{oc}} \right)X_{\text{pc}}+\delta{{}^{13}C}_{\text{oc}}$ | (S76) |
|  |  |

Solving Eq (S75) for $X_{\text{pc}}$ and substituting into Eq. (S76) reveals how $\delta{{}^{13}C}_{\Sigma}$ is expected to change hyperbolically with changes in ${C:N}_{\Sigma}$ as a parametric function of $X_{\text{pc}}.$

|  |  |
| --- | --- |
| $\delta{{}^{13}C}_{\Sigma}=\left( \frac{\delta{{}^{13}C}_{\text{pc}}-\delta{{}^{13}C}_{\text{oc}}}{\frac{1}{{C:N}_{\text{p}}}-\frac{1}{{C:N}_{\text{om}}}} \right)\frac{1}{{C:N}_{\Sigma}}+\left( \frac{\delta{{}^{13}C}_{\text{oc}}\frac{1}{{C:N}_{\text{p}}}-\frac{1}{{C:N}_{\text{om}}}\delta{{}^{13}C}_{\text{pc}}}{\frac{1}{{C:N}_{\text{p}}}-\frac{1}{{C:N}_{\text{om}}}} \right)$ | (S77) |
|  |  |

Thus, we should expect the observed composition a sample ($\delta{{}^{13}C}_{\Sigma}$ and ${C:N}_{\Sigma}$) to hyperbolically approach that of pure plastic ($\delta{{}^{13}C}_{\text{pc}}$ and ${C:N}_{\text{p}}$) as the mole fraction of plastic carbon contamination increases from 0 to 1.

**The relationship between Δ^14^C and C:N in mixtures of MP and natural organic matter**

The Δ^14^C value of a mixture is a linear function of the Δ^14^C values of each endmember and the mole fraction of plastic in the mixture (Medina Faull, 2022).

|  |  |
| --- | --- |
| $\Delta{{}^{14}C}_{\Sigma}=\left( \Delta{{}^{14}C}_{\text{pc}}-\Delta{{}^{14}C}_{\text{oc}} \right)X_{\text{pc}}+\Delta{{}^{14}C}_{\text{oc}}$ | (S78) |
|  |  |

$\Delta{{}^{14}C}_{\Sigma}$ has the same functional dependence on $X_{\text{pc}}$ as $\delta{{}^{13}C}_{\Sigma}$ (Eq. S76), and so can be shown to have a similar hyperbolic relationship with $C:N_{\Sigma}$.

|  |  |
| --- | --- |
| $\Delta{{}^{14}C}_{\Sigma}=\left( \frac{\Delta{{}^{14}C}_{\text{pc}}-\Delta{{}^{14}C}_{\text{oc}}}{\frac{1}{{C:N}_{\text{p}}}-\frac{1}{{C:N}_{\text{om}}}} \right)\frac{1}{{C:N}_{\Sigma}}+\left( \frac{\Delta{{}^{14}C}_{\text{oc}}\frac{1}{{C:N}_{\text{p}}}-\frac{1}{{C:N}_{\text{om}}}\delta{{}^{14}C}_{\text{pc}}}{\frac{1}{{C:N}_{\text{p}}}-\frac{1}{{C:N}_{\text{om}}}} \right)$ | (S79) |
|  |  |

**Error propagation for δ^13^C theoretical relationship:**

Based on eq 76, if we plot $\delta{{}^{13}C}_{\Sigma}$ vs. $X_{\text{pc}}$ the slope is $\delta{{}^{13}C}_{\text{pc}}-\delta{{}^{13}C}_{\text{oc}}$ and the intercept is $\delta{{}^{13}C}_{\text{oc}}$. Therefore, the theoretical uncertainty of the slope is the standard deviation of the average $\delta{{}^{13}C}_{\text{oc}}$ of the sediment. The δ^13^C values in the slope were measured independently and therefore assumed to be uncorrelated. The uncertainty in the slope can be approximated with a Taylor Series expansion in the usual way.

|  |  |
| --- | --- |
| $\sigma_{slope}=\sqrt{\sigma_{\delta{{}^{13}C}_{\text{pc}}}^{2}+\sigma_{\delta{{}^{13}C}_{\text{oc}}}^{2}}$ | (S80) |
|  |  |

NOSAMS reported the δ^13^C precision to be ±0.14 ‰. Therefore, we assigned ±0.14‰ as the uncertainty on the intercept.

**Error propagation for Δ^14^C theoretical relationship:**

Based on eq 78, if we plot of $\Delta{{}^{14}C}_{\Sigma}$ vs. $X_{\text{pc}}$ the slope is $\Delta{{}^{14}C}_{\text{pc}}-\Delta{{}^{14}C}_{\text{oc}}$ and the intercept is $\Delta{{}^{14}C}_{\text{oc}}$. Therefore, the theoretical uncertainty of the slope is the standard deviation of the average $\Delta{{}^{14}C}_{\text{oc}}$ of the sediment. The Δ^14^C values in the slope were measured independently and therefore assumed to be uncorrelated. The uncertainty in the slope can be approximated with a Taylor Series expansion in the usual way.

|  |  |
| --- | --- |
| $\sigma_{slope}=\sqrt{\sigma_{\Delta{{}^{14}C}_{\text{pc}}}^{2}+\sigma_{\Delta{{}^{14}C}_{\text{oc}}}^{2}}$ | (S81) |
|  |  |

**Error propagation for C:N theoretical relationship:**

|  |  |
| --- | --- |
| $\frac{1}{{C:N}_{\Sigma}}=\left( \frac{1}{{C:N}_{\text{p}}}-\frac{1}{{C:N}_{\text{om}}} \right)X_{\text{pc}}+\frac{1}{{C:N}_{\text{om}}}$ | (S82) |
|  |  |

Therefore, solving for C:N, we have

|  |  |
| --- | --- |
| ${C:N}_{\Sigma}=\frac{1}{\left( \frac{1}{{C:N}_{\text{p}}}-\frac{1}{{C:N}_{\text{om}}} \right)X_{\text{pc}}+\frac{1}{{C:N}_{\text{om}}}}$ | (S83) |
|  |  |

The coefficient on $X_{\text{pc}}$ is $\left( \frac{1}{{C:N}_{\text{p}}}-\frac{1}{{C:N}_{\text{om}}} \right)$. This is the hyperbolic slope, $m$.

Therefore, the uncertainty on $m$ is

|  |  |
| --- | --- |
| $\sigma_{m}=\sqrt{\left( \frac{1}{{C:N}_{\text{p}}^{2}} \right)^{2}\sigma_{{C:N}_{\text{p}}}^{2}+\left( \frac{1}{{C:N}_{\text{om}}^{2}} \right)^{2}\sigma_{{C:N}_{\text{om}}}^{2}}$ | (S84) |
|  |  |

And the other constant term, $\frac{1}{{C:N}_{\text{om}}}$, is the hyperbolic intercept, $b$. So, by the same approach, it’s uncertainty is:

|  |  |
| --- | --- |
| $\sigma_{b}=\frac{\sigma_{{C:N}_{\text{om}}}}{{C:N}_{\text{om}}^{2}}$ | (S85) |
|  |  |
