# Supplementary material for: Microplastic contaminants potentially distort our understanding of the ocean’s carbon cycle
Source: PLoS One. 2025 Oct 13;20(10):e0334546. doi: 10.1371/journal.pone.0334546 (PMC12517520; doi:10.1371/journal.pone.0334546)
Supplement: S2 Text — (DOCX) [file pone.0334546.s002.docx]

Supporting Information for

**Microplastic contaminants potentially distort our understanding of the ocean's carbon cycle**

Luis E. Medina Faull* (ID 0000-0002-9425-986X), Gordon T. Taylor (ID 0000-0002-6925-7571), and Steven R. Beaupré (ID 0000-0001-6964-1058)

School of Marine and Atmospheric Sciences, Stony Brook University, New York, U.S.A

**Introduction**

This study examines potential errors imposed by microplastic (MP) contamination on measurements of natural organic matter (OM) during elemental analysis (EA), IRMS and AMS.

Text S2 details the methodology for the experiment designed to test whether a realistic range of MP particle sizes (S1 Table) affects oxidation efficiency in an EA and whether MP particle size affects the degree to which plastic loadings induce measurement errors. This experiment was conducted via EA at the Geosciences Department, Stony Brook University.

**Text S2**

**MP size fraction combustion experiment**

Triplicate PS samples from four different size fractions (60 - 125, 125 – 250, and 250 - 600 µm), were combusted to test the hypothesis that EA combustion efficiency is unaffected by plastic particle size.

**Plastic samples: Source and preparation**

Commercial PS black pellets, approximately 5 mm in diameter, were obtained from Domino Plastics Company Inc, NY, US. Once cleaned with 70 % ethanol, pellets were frozen at -80 °C to facilitate their breakup while grinding to smaller particles using a stainless steel blender (Waring Inc.). The mixture then was size-fractionated sequentially with analytical stainless-steel sieves (DIN 4188, Retsch GmbH, Germany) into particles with nominal diameter ranges of 60 – 125, 125 – 250, and 250 – 600 μm.

**Sediment samples: Source and preparation**

Sediment samples were collected in 1993 at station P, one of 19 Long Island Sound Study sites (Gerino et al., 1998), located in central Long Island Sound (41° 10.03´ N, 72° 57,43´W) at 16 m water depth. Sediments were retrieved using sediment box cores (depth in core 230 – 240 cm) and frozen (-20°C) until the present analysis. Once thawed, 250 g sediment subsamples were homogenized in a porcelain mortar for several minutes. Carbonates and dissolved inorganic carbon were removed by titration with a 10% HCl solution. After reaching a pH of 2–2.5, samples were dried in an oven (60°C) for 48 h and stored as a fine powder at -20°C until further use. This procedure ensured that CO_2_ produced by the samples was derived from biogenic organic carbon and plastic particle amendments (Beaupré et al., 2016).

**Elemental analysis experiment**

Samples were combusted using a Thermo Scientific^TM^ IsoLink^TM^ Elemental Analyzer coupled to a Delta V^TM^ Plus continuous flow isotope ratio mass spectrometer (EA-IRMS) operated by the Geosciences Department, Stony Brook University. Briefly, plastic and sediment samples were weighed on a microbalance (Mettler Toledo, AX105; 0.001 g accuracy). Pure plastic, pure sediment, and admixtures were placed in tin cups and dropped into the oxidation chamber via an autosampler where they burned in an oxygenated atmosphere at greater than 900°C in the presence of an oxidation catalyst. Isotopic signatures of samples are reported relative to CO_2_ derived from Vienna Pee Dee Belemnite carbonate (V-PDB). Instrument drift and precision were monitored by repeat analysis of internationally certified reference standards of Glycine (USGS65, n = 119), Glutamic Acid (IU L-Glutamic Acid, n = 117), and Caffeine (IAEA-600, n = 111), which were run between every five samples.

Total PS carbon content among all samples varied from 0.33 to 0.49 mg C per sample (Table S1). The % C values were not significantly different between the various PS size fractions (Table 1, supplemental information; p > 0.05; Kruskal-Wallis test of significance). Furthermore, % C values did not vary with individual sample masses. Therefore, PS combustion efficiency was statistically independent of particle size and sample mass over the range examined.
