# Supplementary material for: Microplastic contaminants potentially distort our understanding of the ocean’s carbon cycle
Source: PLoS One. 2025 Oct 13;20(10):e0334546. doi: 10.1371/journal.pone.0334546 (PMC12517520; doi:10.1371/journal.pone.0334546)
Supplement: S1 Table — Pa (1,2,3), Pb (1,2,3) and Pc (1,2,3) are triplicate polystyrene samples of different size fractions. Sample mass (MP mass) was measured by microbalance. (DOCX) [file pone.0334546.s003.docx]

| **Table S1**. Combustion performance of EA-IRMS for polystyrene (PS) microparticles within three different size fractions. Pa (1,2,3), Pb (1,2,3) and Pc (1,2,3) are triplicate polystyrene samples of different size fractions. Sample mass (MP mass) was measured by microbalance. | | | | |
| --- | --- | --- | --- | --- |
| **Sample ID** | **Sample mass** (mg PS) | **Yield**  (mg C) | **Yield** (µmol C) | **% Carbon** (% by wt) |
| ***250 – 600 μm size fraction*** | | | | |
| Pa1 | 0.54 | 0.33±0.003 | 43.52±0.44 | 91.72±3.76 |
| Pa2 | 0.38 | 0.52±0.005 | 29.66±0.30 | 95.48±2.53 |
| Pa3 | 0.36 | 0.32±0.003 | 27.11±0.27 | 91.72±3.90 |
| ***125 – 250 μm size fraction*** | | | | |
| Pb1 | 0.36 | 0.33±0.003 | 27.56±0.28 | 91.71±3.76 |
| Pb2 | 0.43 | 0.39±0.004 | 32.88±0.33 | 90.90±3.11 |
| Pb3 | 0.38 | 0.33±0.003 | 29.05±0.29 | 90.07±3.48 |
| ***60 – 125 μm size fraction*** | | | | |
| Pc1 | 0.50 | 0.45±0.005 | 37.32±0.37 | 89.75±2.69 |
| Pc2 | 0.52 | 0.46±0.005 | 38.94±0.39 | 90.56±2.61 |
| Pc3 | 0.39 | 0.34±0.003 | 28.64±0.29 | 89.50±3.49 |
| Sample mass uncertainties were 0.01 mg. | | | | |
