# Supplementary material for: Microplastic contaminants potentially distort our understanding of the ocean’s carbon cycle
Source: PLoS One. 2025 Oct 13;20(10):e0334546. doi: 10.1371/journal.pone.0334546 (PMC12517520; doi:10.1371/journal.pone.0334546)
Supplement: S2 Table — Uncertainties in % plastic mass were propagated from the plastic mass and total mass assuming their uncertainties were ± 1 μg. (DOCX) [file pone.0334546.s004.docx]

| **Table S2.** Masses of pure Polystyrene (PS) microplastics, pure sediment, and their admixtures, and the % plastic by mass in each sample. Uncertainties in % plastic mass were propagated from the plastic mass and total mass assuming their uncertainties were ± 1 μg. | | | | |
| --- | --- | --- | --- | --- |
| **Sample Id** | **Sediment Mass** | **Plastic Mass** | **Total Mass** | **% Plastic mass** |
|  | (µg) | (µg) | (µg) | (μg /μg) |
| ***Pure Microplastics*** | | | | |
| PS1 | 0 | 411 | 411 | 100 |
| PS2 | 0 | 360 | 360 | 100 |
| PS3 | 0 | 430 | 430 | 100 |
| ***Admixtures*** | | | | |
| PS-Sed1 | 5970 | 80 | 6050 | 1.32±0.001 |
| PS-Sed 2 | 5330 | 130 | 5460 | 2.38±0.003 |
| PS-Sed 3 | 5320 | 180 | 5500 | 3.27±0.003 |
| PS-Sed 4 | 5050 | 210 | 5260 | 3.99±0.004 |
| PS-Sed 5 | 5780 | 230 | 6010 | 3.82±0.004 |
| PS-Sed 6 | 5480 | 310 | 5790 | 5.35±0.01 |
| PS-Sed 7 | 6510 | 360 | 6870 | 5.24±0.01 |
| ***Pure Sediments*** | | | | |
| Sed 1 | 6040 | 0 | 6040 | 0 |
| Sed 2 | 5880 | 0 | 5880 | 0 |
| Sed 3 | 7070 | 0 | 7070 | 0 |
