# Supplementary material for: Microplastic contaminants potentially distort our understanding of the ocean’s carbon cycle
Source: PLoS One. 2025 Oct 13;20(10):e0334546. doi: 10.1371/journal.pone.0334546 (PMC12517520; doi:10.1371/journal.pone.0334546)
Supplement: S3 Table — (DOCX) [file pone.0334546.s005.docx]

| **Table S3**. Elemental composition of the pure Polystyrene (PS) microplastics, pure sediment, and admixture samples listed in Table S2, as measured by EA-IRMS. | | | | | | | |
| --- | --- | --- | --- | --- | --- | --- | --- |
| **Sample Id** | **Yield C** | **Yield C** | **% C** | **Yield N** | **Yield N** | **% N** | **C:N** |
|  | (μg C) | (μmol C) | (μg C/μg sed) | (μg N) | (μmol N) | (μg N/μg sed) | (mol/mol) |
| ***Pure Microplastics*** | | | | | | | |
| PS1 | 379±3.79 | 31.60±0.32 | 92.5±2 | 0.00 | 0.00 | 0.00 | — |
| PS2 | 372±3.72 | 31.02±0.31 | 91.7±2 | 0.00 | 0.00 | 0.00 | — |
| PS3 | 384±3.84 | 32.02±0.32 | 90.9±2 | 0.00 | 0.00 | 0.00 | — |
| ***Admixtures*** | | | | | | | |
| PS-Sed1 | 119±1.19 | 9.9±0.10 | 1.2±0.01 | 8.48±0.08 | 0.61±0.06 | 0.0014 | 16.4±0.2 |
| PS-Sed2 | 161±1.61 | 13.4±0.13 | 2.3±0.02 | 7.51±0.08 | 0.54±0.05 | 0.0013 | 25.1±0.4 |
| PS-Sed3 | 198±1.99 | 16.6±0.17 | 2.9±0.03 | 6.38±0.06 | 0.46±0.05 | 0.0011 | 36.3±0.5 |
| PS-Sed4 | 233±2.34 | 19.5±0.20 | 3.8±0.04 | 7.08±0.07 | 0.51±0.05 | 0.0013 | 38.5±0.5 |
| PS-Sed5 | 262±2.63 | 21.9±0.22 | 3.7±0.04 | 7.89±0.08 | 0.56±0.06 | 0.0013 | 38.8±0.5 |
| PS-Sed6 | 333±3.33 | 27.8±0.28 | 3.6±0.04 | 9.03±0.09 | 0.64±0.06 | 0.0011 | 43.1±0.6 |
| PS-Sed7 | 361±3.61 | 30.1±0.30 | 4.6±0.05 | 8.49±0.08 | 0.61±0.06 | 0.0012 | 49.6±0.7 |
| ***Pure Sediments*** | | | | | | | |
| Sed 1 | 43±0.43 | 3.62±0.04 | 0.71±0.007 | 7.50±0.08 | 0.53±0.05 | 0.0011 | 6.8±0.1 |
| Sed 2 | 43±0.44 | 3.63±0.04 | 0.73±0.008 | 7.54±0.08 | 0.54±0.05 | 0.0012 | 6.8±0.1 |
| Sed 3 | 52±0.52 | 4.33±0.04 | 0.73±0.008 | 9.06±0.09 | 0.65±0.07 | 0.0012 | 6.7±0.1 |
