# Supplementary material for: Microplastic contaminants potentially distort our understanding of the ocean’s carbon cycle
Source: PLoS One. 2025 Oct 13;20(10):e0334546. doi: 10.1371/journal.pone.0334546 (PMC12517520; doi:10.1371/journal.pone.0334546)
Supplement: S4 Table — Uncertainties in % plastic mass were propagated from the plastic mass and total mass assuming their uncertainties were ± 1 μg. (DOCX) [file pone.0334546.s006.docx]

| **Table S4.** Masses of pure Polyethylene (PE) microplastics, pure sediment, and their admixtures, and the % plastic by mass in each sample. Uncertainties in % plastic mass were propagated from the plastic mass and total mass assuming their uncertainties were ± 1 μg. | | | | |
| --- | --- | --- | --- | --- |
| **Sample Id  (NOSAMS ID #)** | **Sediment Mass** (µg) | **Plastic Mass** (µg) | **Total Mass** (µg) | **% Plastic mass** (μg /μg) |
| ***Pure Microplastics*** | | | | |
| PE1 (OS-173125) | 0 | 1005 | 1005 | 100 |
| PE2 (OS-173126) | 0 | 1113 | 1113 | 100 |
| PE3 (OS-173127) | 0 | 1094 | 1094 | 100 |
| ***Admixtures*** | | | | |
| PE-S1 (OS-173128) | 10170 | 940 | 11110 | 8.47 ± 0.01 |
| PE-S2 (OS-173129) | 20589 | 868 | 21457 | 4.05 ± 0.005 |
| PE-S3 (OS-173130) | 31373 | 721 | 32094 | 2.25 ± 0.003 |
| PE-S4 (OS-173131) | 40163 | 653 | 40816 | 1.60 ± 0.002 |
| PE-S5 (OS-173132) | 50877 | 508 | 51385 | 0.989 ± 0.002 |
| PE-S6 (OS-173141) | 60713 | 434 | 61147 | 0.710 ± 0.002 |
| PE-S7 (OS-173142) | 70399 | 324 | 70723 | 0.401 ± 0.001 |
| PE-S8 (OS-173143) | 80140 | 214 | 80354 | 0.266 ± 0.001 |
| PE-S9 (OS-173144) | 90127 | 129 | 90256 | 0.143 ± 0.001 |
| ***Pure Sediments*** | | | | |
| Sed 1 (OS-173145) | 100257 | 0 | 100257 | 0 |
| Sed 2 (OS-173146) | 100238 | 0 | 100238 | 0 |
| Sed 3 (OS-173147) | 100209 | 0 | 100209 | 0 |
