# Supplementary material for: Microplastic contaminants potentially distort our understanding of the ocean’s carbon cycle
Source: PLoS One. 2025 Oct 13;20(10):e0334546. doi: 10.1371/journal.pone.0334546 (PMC12517520; doi:10.1371/journal.pone.0334546)
Supplement: S5 Table — (DOCX) [file pone.0334546.s007.docx]

| **Table S5**. Elemental composition of the pure Polyethylene (PE) microplastics, pure sediment, and admixture samples listed in Table S4, as measured by EA-IRMS. | | | | | | | |
| --- | --- | --- | --- | --- | --- | --- | --- |
| **Sample Id** | **Yield C** (μg C) | **Yield C** (μmol C) | **% C** (μg C/μg sed) | **Yield N** (μg N) | **Yield N** (μmol N) | **% N** (μg N/μg sed) | **C:N** (mol/mol) |
| ***Pure Microplastics*** | | | | | | | |
| PE1 | 807±8.07 | 67.20±0.67 | 80.32±1.12 | 0.00 | 0.00 | 0.00 | — |
| PE2 | 897±8.97 | 74.68±0.75 | 80.60±1.07 | 0.00 | 0.00 | 0.00 | — |
| PE3 | 783±7.83 | 65.22±0.65 | 71.61±0.96 | 0.00 | 0.00 | 0.00 | — |
| ***Admixtures*** | | | | | | | |
| PE-S1 | 876±8.75 | 72.95±0.73 | 7.89±0.08 | 12.49±0.12 | 0.83±0.08 | 0.11 | 86.9±1.2 |
| PE-S2 | 926±9.26 | 77.15±0.77 | 4.32±0.04 | 25.19±0.25 | 1.68±0.017 | 0.12 | 45.6±0.6 |
| PE-S3 | 894±8.94 | 74.51±0.75 | 2.79±0.03 | 50.60±0.51 | 3.37±0.034 | 0.16 | 21.9±0.3 |
| PE-S4 | 970±9.70 | 80.81±0.81 | 2.38±0.02 | 52.18±0.52 | 3.48±0.035 | 0.13 | 23.0±0.3 |
| PE-S5 | 1093±10.93 | 91.01±0.91 | 2.13±0.02 | 76.38±0.76 | 5.09±0.051 | 0.15 | 17.7±0.3 |
| PE-S6 | 996±9.96 | 82.99±0.83 | 1.63±0.02 | 74.87±0.75 | 4.99±0.050 | 0.12 | 16.5±0.2 |
| PE-S7 | 1124±11.24 | 93.59±0.94 | 1.59±0.02 | 87.79±0.88 | 5.85±0.059 | 0.12 | 15.9±0.2 |
| PE-S8 | 1215±12.15 | 101.19±1.02 | 1.51±0.02 | 102.28±1.02 | 6.82±0.068 | 0.13 | 14.7±0.2 |
| PE-S9 | 1233±12.33 | 102.69±1.03 | 1.37±0.01 | 114.60±1.11 | 7.64±0.076 | 0.13 | 13.3±0.2 |
| ***Pure Sediments*** | | | | | | | |
| Sed 1 | 1136±11.36 | 94.59±0.95 | 1.13±0.01 | 122.26±1.22 | 8.15±0.082 | 0.12 | 11.5±0.2 |
| Sed 2 | 1108±11.08 | 92.29±0.92 | 1.11±0.01 | 121.67±1.22 | 8.11±0.081 | 0.12 | 11.3±0.2 |
| Sed 3 | 1118±11.18 | 93.09±0.93 | 1.12±0.01 | 127.41±1.27 | 8.49±0.085 | 0.13 | 10.9±0.2 |
