# Supplementary material for: Microplastic contaminants potentially distort our understanding of the ocean’s carbon cycle
Source: PLoS One. 2025 Oct 13;20(10):e0334546. doi: 10.1371/journal.pone.0334546 (PMC12517520; doi:10.1371/journal.pone.0334546)
Supplement: S6 Table — (DOCX) [file pone.0334546.s008.docx]

| **Table S6.** Carbon isotopic data measured at the National Ocean Sciences Accelerator Mass spectrometry (NOSAMS) for pure PE microparticles, admixtures, and sediment. | | | | |
| --- | --- | --- | --- | --- |
| **Sample ID** | **F Modern** | **Age** (^14^C yr) | **Δ^14^C** (‰) | **δ^13^C** (‰) |
| ***Pure Microplastics*** | | | | |
| PE 1 | 0.0084±0.0020 | 38400±1900* | -991±2 | -31.49±0.14 |
| PE 2 | 0.0040±0.0020 | 44300±4000* | -996±2 | -31.39±0.14 |
| PE 3 | 0.0086±0.0020 | 38200±1900* | -991±2 | -30.97±0.14 |
| ***Admixtures*** | | | | |
| PE-S 1 | 0.0951±0.0019 | 18900±160 | -905±1.9 | -30.02±0.14 |
| PE-S 2 | 0.1665±0.0018 | 14400±85 | -834±1.8 | -28.59±0.14 |
| PE-S 3 | 0.2379±0.0018 | 11550±60 | -764±1.8 | -27.03±0.14 |
| PE-S 4 | 0.3018±0.0016 | 9620±45 | -700±1.6 | -26.8±0.14 |
| PE-S 5 | 0.4090±0.0015 | 7180±30 | -594±1.5 | -25.28±0.14 |
| PE-S 6 | 0.4450±0.0016 | 6510±30 | -559±1.6 | -24.95±0.14 |
| PE-S 7 | 0.5148±0.0014 | 5330±20 | -489±1.4 | -23.9±0.14 |
| PE-S 8 | 0.5547±0.0016 | 4740±25 | -450±1.6 | -23.23±0.14 |
| PE-S 9 | 0.5885±0.0014 | 4260±20 | -416±1.4 | -22.46±0.14 |
| ***Pure Sediments*** | | | | |
| Sed 1 | 0.641±0.0014 | 3570±20 | -364±2 | -21.63±0.14 |
| Sed 2 | 0.641±0.0015 | 3570±20 | -364±2 | -21.65±0.14 |
| Sed 3 | 0.641±0.0015 | 3570±20 | -364±2 | -21.62±0.14 |

- Data suggested that pure MPs appeared younger than predicted, i.e., not completely devoid of ^14^C (eq 78). A potential explanation could be attributed to additives (pigments, plasticizers) added to plastic and containing modern carbon. Raman analysis of the plastic fragments confirmed a chemical structure of Polyethylene (PE); however, when compared with a reference PE Raman spectrum, several extra diagnostic peaks appeared, potentially indicating the presence of these additives.
